# Clinical phenotyping of asthma patients with elevated sputum eosinophils and low blood eosinophils: a post-hoc analysis of the multicentre ATLANTIS cohort

**DOI:** 10.1016/j.ebiom.2026.106366

**Published:** 2026-07-11

**Authors:** Pauline J.M. Kuks, Atheer M.A. Aabed, Lauren C.A. Premereur, Monica Kraft, Salman Siddiqui, Leonardo M. Fabbri, Bianca Beghé, Klaus F. Rabe, Alberto Papi, Christopher E. Brightling, Dave Singh, Alessio Piraino, Ulrica Scaffidi-Argentina, Janwillem H. Kocks, Lies Lahousse, Huib A.M. Kerstjens, Irene H. Heijink, Simon D. Pouwels, Dirk-Jan Slebos, Maarten van den Berge

**Affiliations:** a Department of Pulmonary Diseases, University of Groningen, University Medical Center Groningen, Groningen, the Netherlands; b University of Groningen, University Medical Center Groningen, Groningen Research Institute for Asthma and COPD, Groningen, the Netherlands; c Samuel Bronfman Department of Medicine, Icahn School of Medicine, Mount Sinai Medical Center, New York, NY, USA; d National Heart & Lung Institute, Imperial College London, London, UK; e Section of Respiratory Medicine, Department of Translational Medicine, University of Ferrara, Italy; f Department of Respiratory Diseases, University of Modena and Reggio Emilia, Modena, Italy; g Department of Medicine, Christian Albrechts University Kiel, Kiel and Lungen Clinic, Grosshansdorf, Germany; h Research Centre on Asthma and COPD, University of Ferrara, Ferrara, Italy; i Institute for Lung Health, National Institute for Health Research Biomedical Research Centre, University of Leicester, Leicester, UK; j Centre for Respiratory Medicine and Allergy, University Hospital of South Manchester, University of Manchester, Manchester, UK; k Global Medical Affairs, Chiesi Farmaceutici S.p.A. Parma, Italy; l Department of Bioanalysis, Ghent University, Ghent, Belgium; m Department of Epidemiology, Erasmus Medical Center, Rotterdam, the Netherlands; n Department of Pathology & Medical Biology, University of Groningen, University Medical Center Groningen, the Netherlands

**Keywords:** Asthma, Eosinophilic asthma, Isolated sputum eosinophilia, Large and small airways disease

## Abstract

**Background:**

Patients with eosinophilic asthma are responsive to treatment with corticosteroids and biologics. Eosinophilia is usually identified based on blood eosinophil counts, but there is discordance between blood and sputum in some cases. A subset of patients may have sputum eosinophilia despite low blood eosinophil levels. The clinical implications of this so-called isolated sputum eosinophilia are unknown. The aim of this study is to investigate the clinical expression of asthma in patients with isolated sputum eosinophilia.

**Methods:**

In this post-hoc ATLANTIS analysis we included patients with available blood and/or sputum data from ATLANTIS. Patients were classified according to blood eosinophils (< or ≥300 cells/μL). Patients with isolated sputum eosinophilia were compared with those with low eosinophils in both compartments.

**Findings:**

Of the 487 patients with low blood eosinophils counts, sputum samples were available in 146. Among these, 25 (17%) had isolated sputum eosinophilia. Compared with patients with low eosinophils in both compartments (n = 121), patients with isolated sputum eosinophilia had more airflow obstruction (*FEV*_*1*_*/FVC ratios (68.8%* vs. *75.4%,* p < *0.01))* and small airways dysfunction *(S*_*cond*_*0.05* vs. *0.03 1/L,* p = *0.02)* and more frequently reported exacerbations in the year prior to inclusion *(28%* vs. *9%,* p = *0.01)*. Clinical characteristics were broadly comparable to those observed in patients with blood eosinophilia.

**Interpretation:**

Isolated sputum eosinophilia occurs in a subgroup of patients with asthma, in association with worse clinical outcomes. These findings suggest that airway eosinophilia may not always be captured by conventional blood biomarkers and warrant further investigation into the clinical implications of this phenotype to determine whether these patients may benefit from treatment strategies targeting type 2 inflammation, such as intensified steroids or biologics.

**Funding:**

Chiesi Farmaceutici sponsored the ATLANTIS study.

**Research in context**

**Evidence before this study**

In preparation for this manuscript, we have searched PubMed studies in English up to March 2026. The following terms were used separately or in combination: “Asthma”, “Blood eosinophils”, “Sputum eosinophils”, “biomarkers”, “Type 2 inflammation”, “FeNO”, “inflammatory profile”, “biologics”, “corticosteroids”. We limited our search to human studies. We assessed the quality of each study based on sample size, use of validated outcomes, and reproducibility.

Previous studies reported that patients with eosinophilic asthma respond better to corticosteroids and biologics than those without eosinophilia. In daily clinical practice, blood counts are generally used to assess eosinophilia. However, sputum is the golden standard and blood eosinophil counts do not always reflect sputum eosinophils. Thus, isolated sputum eosinophilia, defined as low blood eosinophils and high eosinophils in sputum may occur in a subset of patients with uncontrolled persistent asthma. So far, the clinical implications of isolated sputum eosinophilia are unknown.

**Added value of this study**

We show that a considerable proportion of patients (17%) with low blood eosinophils exhibit isolated sputum eosinophilia. Our study demonstrates that patients with isolated sputum eosinophilia exhibit worse clinical outcomes, including more severe airflow obstruction, small airways dysfunction, thicker airway walls on CT, and a higher likelihood of prior exacerbations.

**Implications of all the available evidence**

Our findings underscore the importance of directly assessing airway eosinophils in patients with uncontrolled asthma when they have low blood eosinophil counts, because those might benefit from intensified steroid or biologics.

## Introduction

Asthma is a heterogeneous chronic respiratory disease characterised by airway inflammation, variable airflow obstruction, and bronchial hyperresponsiveness.^1^ Despite some common clinical features, asthma encompasses distinct phenotypes with different observable clinical characteristics, and endotypes with markers reflecting different underlying pathophysiological mechanisms.^2^ Recognising this heterogeneity is of importance, as treatment response varies across different asthma subtypes.^1^^,^^2^

Asthma endotyping is commonly based on the dominant airway inflammatory pattern, most notably distinguishing type 2-high from type 2-low asthma. Patients with type 2-high eosinophilic asthma are more likely to respond effectively to intensified treatment with inhaled corticosteroids and/or biologics targeting interleukin-5 (IL-5), IL-4/IL-13, or their receptors.^3^^,^^4^ Therefore, the identification of type 2-high inflammation plays an important role in guiding treatment. Type 2-high inflammation is typically defined by elevated levels of Fractional exhaled Nitric Oxide (FeNO) and/or eosinophils in sputum or blood. Commonly used thresholds include FeNO ≥25 parts per billion (ppb),^5^ sputum eosinophils ≥2–3% of non-squamous cells, and blood eosinophils ≥150–300 cells/μL.^6^, ^7^, ^8^, ^9^ While sputum eosinophil counts are considered the golden standard for diagnosing type 2-high eosinophilic asthma, sputum induction, processing and analysis are labour-intensive, and often unavailable in routine clinical settings.^10^ As a result, eosinophilia in asthma is typically assessed using blood eosinophil counts. Although studies have reported an association between blood and sputum eosinophil levels,^11^^,^^12^ the strength of the correlation varies between studies and is generally modest to weak.^13^^,^^14^

Thus, some patients show discordance between blood and sputum eosinophilia, resulting in a subgroup with high sputum eosinophils despite low blood cell counts. The clinical implications of this so-called isolated sputum eosinophilia in asthma are unknown. It is possible that these patients represent a subgroup with clinically relevant type 2-high inflammation, associated with more severe disease compared to those with low eosinophils in both compartments. If so, these patients with isolated sputum eosinophilia may be misclassified as having type 2-low disease, potentially depriving them of treatment strategies targeted at type 2-high inflammation.

Despite the potential clinical relevance, it has not been studied before whether patients with isolated sputum eosinophilia, defined as elevated sputum eosinophils without elevated blood eosinophil counts, have a more severe or a different disease expression compared with patients who have low eosinophils in both compartments. In addition, we explored whether patients with isolated sputum eosinophilia exhibit clinical characteristics comparable to those with elevated blood eosinophils.

To investigate this, we performed a post-hoc analysis within the ATLANTIS (AssessmenT of SmalL Airways involvement In aSthma) study, a large multicentre observational study focused on understanding the role of the small airways in asthma.^15^^,^^16^

## Methods

### Participants

The ATLANTIS study (NCT02123667) is a large multicentre observational cohort study conducted between June 2014 and March 2017 across 29 primary and secondary care centres in nine countries (Brazil, Canada, China, Germany, Italy, the Netherlands, Spain, the United Kingdom, the United States of America). The protocol and inclusion and exclusion criteria can be found on www.clinicaltrials.gov (NCT02123667) *and in the* Supplementary Materials. Participants were adults aged 18–65 years with physician-diagnosed asthma, confirmed by objective evidence of bronchodilator reversibility and/or airway hyperresponsiveness. Patients with asthma, across all severities, on any previous regular asthma treatment (rescue β2-agonists alone included) at a stable dose, for a minimum of 8 weeks prior to baseline visit, were included. All patients were required to have clinically stable asthma at study entry, defined as the absence of exacerbations. Individuals with a diagnosis of chronic obstructive pulmonary disease (COPD) or a smoking history of ≥10 pack-years were excluded. Participants were recruited through general practitioners, chest physicians’ databases, and public advertisements across participating centres.

### Ethics

The study protocol was approved by the local medical ethics committees of all participating centres, and written informed consent was obtained from all participants.

### Study design and procedures

All data were collected as part of the ATLANTIS study according to the standardised study protocol by trained personnel at participating centres. The Supplementary Materials includes the full study protocol. Briefly, at baseline, participants were extensively characterised, including the assessment of asthma control using the Asthma Control Questionnaire (ACQ6) and the measurements of FeNO. Sex was self-reported by study participants at study inclusion. Large airways function was assessed using spirometry, and small airways function was evaluated using impulse oscillometry (IOS) and multiple breath nitrogen washout (MBNW). Bronchial hyperresponsiveness was assessed with a methacholine challenge test, and computed tomography (CT) scans were performed to evaluate airway wall thickness and lung density. In addition, blood and sputum samples were collected. Sputum was collected in a subset of participants (see Supplementary Table S8) at selected secondary care centres only in Europe, the United States, and Canada. Plugs of the induced sputum were selected to ensure absence of squamous cells and presence of lower respiratory tract cells. The sputum samples were processed as described by Hargreave et al.,^17^ and percentages of non-squamous cells were counted. Participants were followed for 12 months, with in-person assessments at 6 and 12 months and interim telephone follow-ups at 3 and 9 months to monitor exacerbations. Exacerbations were defined as asthma worsening requiring ≥3 days of systemic corticosteroids, an emergency department visit, or hospitalisation.

### Cut-offs to define eosinophilia

The current ATLANTIS post-hoc analysis included subjects without blood eosinophilia and available sputum samples, as well as all patients with blood eosinophilia. Patients with low blood eosinophil counts but without sputum counts available were excluded from this analysis. Blood eosinophilia was defined as ≥300 cells/μL whereas sputum eosinophilia as ≥2%, consistent with recommended lower thresholds of 2–3%. Patients with blood eosinophil counts <300 cells/μL (7) were selected and stratified into two groups: those with isolated sputum eosinophilia and those with low sputum eosinophils in both compartments. Given that FeNO is also a conventional biomarker of type 2-high inflammation, we performed an additional analysis restricted to patients with both low blood eosinophils and low FeNO (≤25 parts per billion (ppb))^5^ to evaluate whether the findings in patients with isolated sputum eosinophilia were robust. Furthermore, given the lack of consensus on a universal definition of eosinophilia, additional sub-analyses were conducted using alternative thresholds reported in the literature. These included blood eosinophil counts <300 or <150 cells/μL combined with sputum eosinophil thresholds of ≥2% or ≥3%.^6^, ^7^, ^8^, ^9^

### Outcomes

The primary outcome of this analysis was the difference in clinical and physiological characteristics between patients with isolated sputum eosinophilia and patients with low eosinophils in both compartments. These characteristics included lung function parameters (FEV_1_, FEV_1_/FVC), measures of small airways dysfunction (including S_COND_ (*ventilation heterogeneity in the conductive zone of the lungs corrected for tidal volume) values)* and R5–R20, and CT-derived airway structural parameters.

Secondary outcomes included the occurrence of asthma exacerbations during the 12-month follow-up period and exacerbations in the year prior to study inclusion. In addition, associations between sputum eosinophil percentages (analysed as a continuous variable) and clinical, physiological, and imaging parameters were evaluated in patients with low blood eosinophil levels.

### Statistics

All statistical analyses were conducted using RStudio (version 2024.12.1.563). Normality was assessed using histograms. Depending on the type and distribution of data, group comparisons were performed using chi-squared tests, independent t-tests, or Mann–Whitney U tests. Baseline characteristics were summarised using the *TableOne* package (version 0.13.2). Non-normally distributed continuous variables were reported as medians with interquartile ranges (IQR). For continuous variables effect estimates and for odds ratios for binary variables are reported as the difference between groups with corresponding 95% confidence intervals. For normally distributed variables, this represents the difference in means, whereas for non-normally distributed variables the Hodges–Lehmann median difference is reported.

Time-to-event analyses for exacerbations were conducted using *survival* (version 3.8.3) and visualised using the *survminer* (version 0.5.0) and *ggplot2* (version 3.5.1) packages. Participants were censored at the time of their first exacerbation or at their final follow-up visit, whichever occurred first.

Multivariable Cox proportional hazards models were adjusted for age, sex, smoking exposure (pack-years), smoking status (current and former smoking), and blood eosinophil counts.

No formal correction for multiple testing was applied, as this analysis was exploratory in nature. Missing data were not imputed. Analyses were performed using available-case data, and patients without sputum measurements were excluded from analyses requiring sputum variables. In addition, sex-disaggregated analyses were performed by repeating the main analyses separately in male and female participants.

### Sample size justification

Since this was a post-hoc analysis using available ATLANTIS data, no formal power calculation was performed.

### Role of funders

Chiesi Farmaceutici sponsored the data collection within ATLANTIS study but did not provide financial support to this post-hoc analysis. The funders did not have a role in the data analyses, interpretation or writing of the report.

## Results

**Fig. 1:**
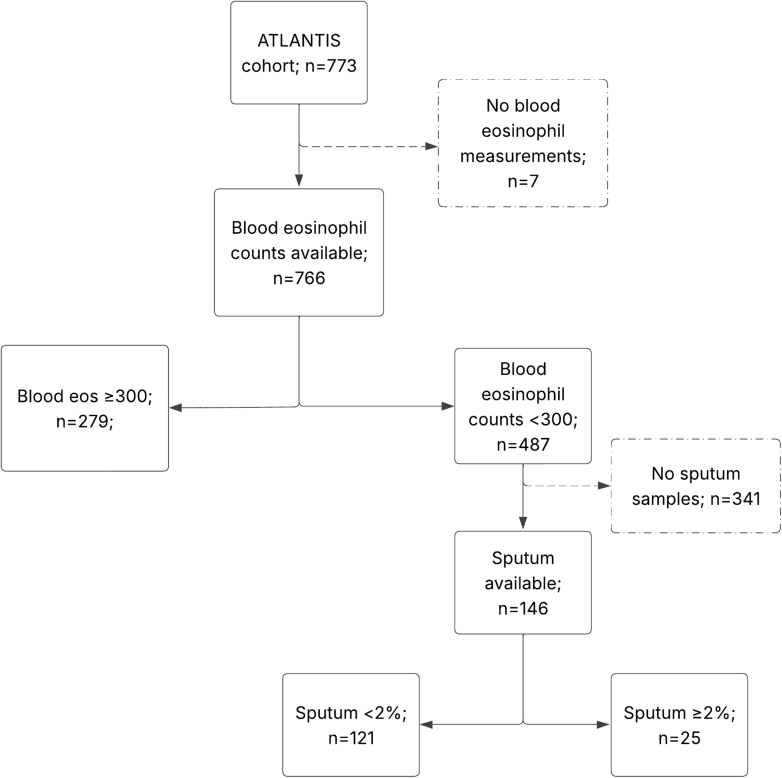
**Flowchart of study population selectionin the ATLANTIS cohort.** ATLANTIS asthma patients were first divided into two groups based on blood eosinophil counts: with (≥300 cells/μL) and without (<300 cells/μL) blood eosinophilia. Among patients without blood eosinophilia, availability of sputum samples was evaluated. In those with available sputum samples, patients were further classified based on sputum eosinophil percentages into groups with sputum eosinophilia (≥2%) and without (<2%) sputum eosinophilia.

Of the 766 patients with asthma included in the ATLANTIS study with blood sampling, 279 subjects had blood eosinophilia (58% female, mean ± SD 43.7 ± 13.5), while 487 did not. Among the 487 patients without blood eosinophilia, sputum samples were available in 146 patients. Among these, 25 (17%) exhibited sputum eosinophilia (40% female, mean ± SD age 47 ± 13 years), compared to 121 (83%) without sputum eosinophilia (57% female, age 44 ± 14 years) (Fig. 1).

**Fig. 2:**
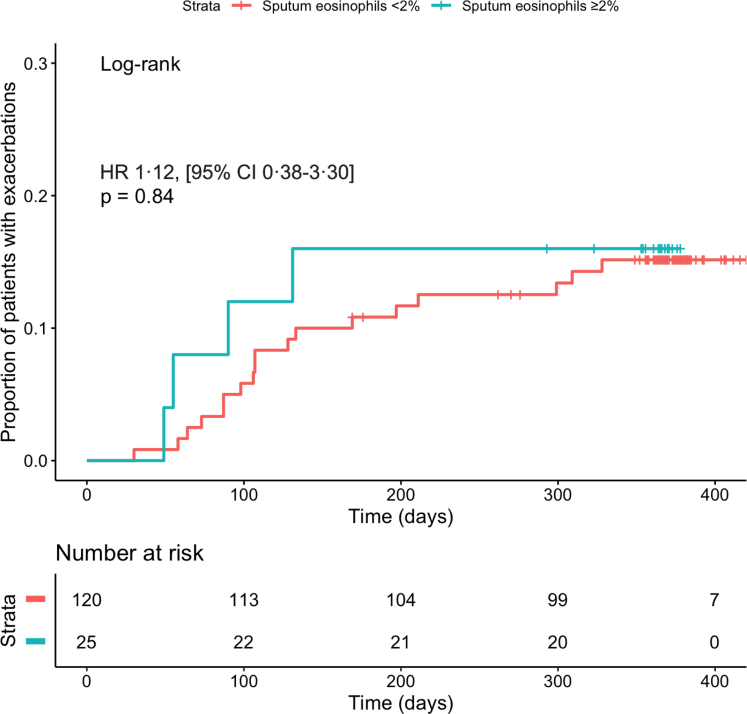
**Time to first exacerbation during the 1-year follow-up in patients with low blood eosinophils (<300 cells/μL) stratified for sputum eosinophilia.** The occurrence of exacerbations in the 1-year follow-up did not statistically significantly differ between those with isolated sputum eosinophilia and those without sputum eosinophilia independently of eosinophils in blood.

Patients with isolated sputum eosinophilia were compared to those without eosinophilia in blood or sputum. Table 1 presents the baseline demographic characteristics of the study population. Clinical and physiological outcomes are presented separately in Table 2. Sputum eosinophil percentages were statistically significant higher in patients with isolated sputum eosinophilia (median 6.5% vs. 0.10%, diff +6.00, [95% CI 4.50, 9.70], p < 0.001), underscoring the discrepancy between blood and sputum eosinophils. Patients with isolated sputum eosinophilia had statistically significant higher median blood eosinophil counts and more pack-years of smoking. These patients also had statistically significant more severe airflow obstruction as reflected by lower FEV_1_/FVC ratios both pre-bronchodilator (64.5% vs. 70.8%, diff −0.06, [95% CI −0.11, −0.02], p = 0.003) and post-bronchodilator (68.8% vs. 75.4%, diff −6.6, [95% CI −12.78, −2.67], p = 0.001), and a trend towards lower pre-bronchodilator FEV_1_% predicted (80.0% vs. 86.8%, diff −6.73, [95% CI −14.94, 1.48], p = 0.051). Patients with isolated sputum eosinophilia also exhibited more severe small airways dysfunction, measured by S_COND_ (0.05 vs. 0.03, diff +0.02, [95% CI −0.01, 0.05], p = 0.020), though no statistically significant differences were observed in other small airways function parameters. On CT, patients with isolated sputum eosinophilia had thicker airway walls (37.3 mm^2^ vs. 32.9 mm^2^, diff +4.40, [95% CI 1.19, 7.61], p = 0.021). In addition, a statistically significant higher proportion of patients with isolated sputum eosinophilia experienced an exacerbation in the year prior to inclusion (28% vs. 9%, OR 3.89, [95% CI 1.29, 11.2], p = 0.013). During the one-year follow-up, 20% of patients with sputum eosinophilia experienced an exacerbation, compared to 16% of patients without sputum eosinophilia; Kaplan–Meier analysis showed no statistically significant difference in time to first exacerbation between the two groups (HR 1.12, [95% CI 0.38, 3.30], p = 0.84) (Fig. 2). In a multivariable Cox proportional hazards model adjusted for age, sex, pack-years in current and former smokers, blood eosinophils, isolated sputum eosinophilia was likewise not statistically significantly associated with time to first exacerbation (HR 3.70, [95% CI 0.13, 10.85], p = 0.45).

**Table 1:** Baseline demographic and clinical characteristics of asthma subjects in ATLANTIS without blood eosinophilia (<300 cells/μL blood) stratified for sputum eosinophilia (≥2%).

	Patients without sputum eosinophilia (<2%)	Patients with sputum eosinophilia (≥2%)
n	121	25
Age, years	44.4 ± 13.5	46.6 ± 13.3
Female sex	69 (57)	10 (40)
BMI, kg/m^2^	28.2 ± 6.07	26.1 ± 4.97
Smoking status		
Never smoker	94 (78)	17 (68)
Former smoker	24 (20)	8 (32)
Current Smoker	3 (2)	0 (0)
Pack-years in current and former smokers	2.70 [1.40, 5.00]	7.10 [5.00, 9.00]
Ethnicity		
Asian	2 (2)	0 (0)
Black or African American	2 (2)	1 (4)
More than one Race	1 (1)	0 (0)
Other	1 (1)	0 (0)
White/Caucasian	115 (94)	24 (96)
GINA classification		
1	33 (27)	4 (16)
2	16 (13)	2 (8)
3	31 (26)	6 (24)
4	39 (32)	11 (44)
5	2 (2)	2 (8)
Use of ICS or ICS/LABA	85 (70)	21 (84)
Daily ICS dose (beclomethasone equivalent) in those on ICS or ICS/LABA, μg	700 [400, 1000]	800 [450, 1000]
Systemic corticosteroids use	2 (2)	2 (8)
ACQ6 score	0.80 [0.17, 1.66]	0.67 [0.33, 1.16]
Age of asthma diagnosis, years	22.0 [10.0, 37.7]	28.0 [7.64, 41.3]
Duration of asthma, years	20.1 [7.55, 39.4]	17.6 [10.9, 22.6]

Data are presented as n, n (%), mean ± SD or median [interquartile range]. BMI = Body Mass Index. GINA = Global Initiative for Asthma (2012 guidelines). ICS = inhaled corticosteroids. ICS/LABA = /long acting beta2 agonist. ACQ6 = asthma control questionnaire 6.

**Table 2:** Clinical outcomes in asthma subjects in ATLANTIS without blood eosinophilia (<300 cells/μL blood) stratified for sputum eosinophilia (≥2%).

	Patients without sputum eosinophilia (<2%)	Patients with sputum eosinophilia (≥2%)	Estimate of effect	95% CI	p-value
n	121	25			
Eosinophils sputum, % of non-squamous cells	0.10 [0.00, 0.40]	6.50 [3.90, 12.90]	Diff +6.00	4.50, 9.70	<0.001
Blood eosinophil count, cells/μL	150 [100, 200]	190 [160, 240]	Diff +0.04	0.01, 0.07	0.0080
Patients with ≥1 exacerbations in the year prior to inclusion, n (%)	11 (9)	7 (28)	OR 3.89	1.29, 11.27	0.013
Positive specific IgE blood screening (Phadiatop test)	76 (75)	19 (91)	OR 3.12	0.83, 20.48	0.14
FeNO, parts per billion	18.0 [12.0, 26.0]	25.5 [17.5, 38.3]	Diff +7.00	2.00, 13.00	0.0080
Pulmonary physiology					
Airway hyperresponsiveness, category					0.69
Very mild (PC_20_ ≥ 4 & <16 mg/ml, PD_20_ ≥ 0.5 & <2 mg)	31 (35)	9 (43)			
Mild (PC_20_ ≥ 1 & <4 mg/ml, PD20 ≥ 0.13 & <0.5 mg)	33 (37)	5 (24)			
Moderate (PC_20_ ≥ 0.25 & <1 mg/ml, PD_20_ ≥ 0.03 & <0.13 mg)	20 (22)	6 (28)			
Severe (PC_20_ < 0.25 mg/ml, PD20 < 0.03 mg)	5 (6)	1 (5)			
FEV_1_% of predicted normal value (pre-bronchodilator)	86.8 (14.8)	80.0 (18.9)	Diff −6.73	−14.94, 1.48	0.051
FEV_1_/FVC (pre-bronchodilator)	70.8 (9.48)	64.5 (9.69)	Diff −0.06	−0.11, −0.02	0.0030
FEV_1_, % predicted (post-bronchodilator)	95.3 (12.8)	90.6 (17.8)	Diff −4.7	−12.31, 3.02	0.13
FEV_1_/FVC, % (post-bronchodilator)	75.4 (8.61)	68.8 (9.93)	Diff −6.6	−12.78, −2.67	0.0010
FEV_1_ reversibility	11.4 (11.0)	14.9 (13.1)	Diff +3.5	−2.2, 9.2	0.16
Residual volume/total lung capacity, % (post-bronchodilator)	31.0 (8.00)	34.0 (8.00)	Diff +3.0	−6.0, 1.00	0.12
R5-20 (post-bronchodilator), kPa/L/s	0.04 [0.02, 0.08]	0.03 [0.00, 0.05]	Diff −0.02	−0.01, 0.05	0.17
S_COND_, 1/L (post-bronchodilator)	0.03 [0.02, 0.04]	0.05 [0.03, 0.06]	Diff +0.02	0.00, 0.03	0.020
S_ACIN_, 1/L (post-bronchodilator)	0.08 [0.06, 0.14]	0.15 [0.07, 0.16]	Diff +0.03	−0.08, 0.01	0.10
CT scan derived parameters					
Voxel index at-950HU	5.22 ± 4.78	4.50 ± 3.58	Diff −0.72	−2.41, 0.97	0.62
Pi10	7.37 ± 1.28	6.99 ± 1.47	Diff −0.38	−1.01, 0.25	0.36
Median lumen area, mm^2^	20.0 ± 4.25	21.3 ± 4.36	Diff +1.30	−0.62, 3.22	0.35
Median airway wall area, mm^2^	32.9 ± 5.45	37.3 ± 7.37	Diff +4.40	1.19, 7.61	0.021
Median total area, mm^2^	53.6 ± 8.92	58.7 ± 11.01	Diff +5.10	0.30, 9.90	0.091
Wall area/total area, %	62.0 ± 3.44	63.7 ± 3.23	Diff +1.70	0.24, 3.16	0.12
Median lung density ratio, E/I	0.81 ± 0.08	0.84 ± 0.07	Diff +0.03	0.00, 0.06	0.28
Lung volume ratio, E/I	0.50 ± 0.14	0.52 ± 0.13	Diff +0.02	0.00, 0.06	0.57
Sputum-derived parameters					
Lymphocytes sputum, % of non-squamous cells	0.60 [0.30, 1.30]	0.60 [0.10, 1.60]	Diff −0.1	−0.4, 0.2	0.53
Macrophages sputum, % of non-squamous cells	44.3 [20.6, 63.6]	33.1 [18.3, 55.5]	Diff −4.8	−17, 5.2	0.31
Neutrophils sputum, % of non-squamous cells	50.4 [28.7, 75.2]	46.9 [21.2, 59.1]	Diff −6.5	−18.8, 5.5	0.25
Blood neutrophil count, cells/μL	3.50 [3.00, 4.55]	3.47 [2.62, 4.10]	Diff −0.28	−0.76, 0.27	0.32

Data are presented as n, n (%), mean ± SD or median [interquartile range]. Effect estimates are reported as mean differences or Hodges–Lehmann median differences for continuous variables, and as odds ratios for binary variables, with corresponding 95% confidence intervals. CI = Confidence Interval. FeNO = fractional exhaled nitric oxide. PC_20_ = provocative concentration that causes a 20% decrease in FEV_1_ from baseline during methacholine challenge. PD_20_ = provocative dose that causes a 20% decrease in FEV_1_ from baseline during methacholine challenge. FEV_1_ = Forced expiratory volume in 1 s. FVC = forced vital capacity. Reversibility FEV_1_ (%) = percent change from initial FEV_1_ after administration of 4 × 100 μg salbutamol, calculated as: ((post-bronchodilator FEV_1_ – pre-bronchodilator FEV_1_)/pre-bronchodilator FEV_1_) × 100. RV/TLC = Residual volume/total lung capacity. R5-R20 = resistance at 5 Hz–resistance at 20 Hz. S_ACIN_ = ventilation homogeneity of the acinar zone of the lungs corrected for tidal volume. S_COND_ = ventilation heterogeneity in the conductive zone of the lungs corrected for tidal volume. Pi10 = 10 mm internal luminal perimeter.

To explore whether patients with isolated sputum eosinophilia represent a distinct clinical phenotype or are comparable to patients with blood eosinophilia (Table 3), the conventional type 2-high biomarkers used in clinical practice, we compared their clinical characteristics, lung function and imaging findings to conventionally identified patients with blood eosinophilia. Those with isolated sputum eosinophilia were less likely to be female and had lower R5-R20 values compared to those with blood eosinophilia. Other clinical characteristics were similar between the groups, with no statistical significant differences in asthma control, lung function, CT scan parameters or exacerbation rates (Table 4).

**Table 3:** Baseline demographic and clinical characteristics of asthma subjects in ATLANTIS asthma patients with blood eosinophilia (≥300 cells/μL blood) and those with isolated sputum eosinophilia (≥2%).

	Patients with blood eosinophilia (≥300 cells/μL)	Patients with sputum eosinophilia (≥2%)
n	279	25
Age, years	43.7 ± 13.5	46.6 ± 13.3
Female sex	161 (57.7)	10 (40)
BMI, kg/m^2^	26.75 ± 5.78	26.1 ± 4.97
Smoking status		
Never smoker	216 (77.4)	17 (68)
Former smoker	53 (19.0)	8 (32)
Current Smoker	10 (3.6)	0 (0)
Pack-years in current and former smokers	4.00 [1.35, 7.50]	7.10 [5.00, 9.00]
GINA classification		
1	39 (14)	4 (16)
2	25 (9)	2 (8)
3	64 (23)	6 (24)
4	127 (45)	11 (44)
5	24 (9)	2 (8)
Use of ICS or ICS/LABA	240 (86)	21 (84)
Daily ICS dose (beclomethasone equivalent) in those on ICS or ICS/LABA, μg	800 [400, 1000]	800 [450, 1000]
Systemic corticosteroids use	9 (3)	2 (8)
Systemic corticosteroids dose (prednisone equivalent) in those on systemic corticosteroids, mg	5.00 [3.50, 12.50]	12.5 [8.8, 16.3]
ACQ6 score	0.83 [0.33, 1.50]	0.67 [0.33, 1.16]
Age of asthma diagnosis, years	22.0 [6.72, 40.8]	28.0 [7.64, 41.3]
Duration of asthma, years	19.4 [6.64, 29.7]	17.6 [10.9, 22.6]

Data are presented as n, n (%), mean ± SD or median [interquartile range]. BMI = Body Mass Index. GINA = Global Initiative for Asthma (2012 guidelines). ICS = inhaled corticosteroids. ICS/LABA = /long acting beta2 agonist. ACQ6 = asthma control questionnaire 6.

**Table 4:** Clinical outcomes in asthma subjects in ATLANTIS stratified for patients with blood eosinophilia (≥300 cells/μL blood) and those with isolated sputum eosinophilia (≥2%).

	Patients with blood eosinophilia (≥300 cells/μL)	Patients with sputum eosinophilia (≥2%)	Estimate of effect	95% CI	p-value
n	279	25			
Eosinophils sputum, % of non-squamous cells	2.20 [0.50, 11.1]	6.50 [3.90, 12.90]	Diff +3.20	1.60, 5.40	0.003
Blood eosinophil count, cells/μL	430 [360, 600]	190 [160, 240]	Diff −0.25	−0.32, −0.20	<0.001
Patients with ≥1 exacerbations in the year prior to inclusion, n (%)	47 (17)	7 (28)	OR +1.92	0.71, 4.68	0.26
Positive specific IgE blood screening (Phadiatop test)	172 (81)	19 (91)	OR +2.26	0.62, 14.57	0.42
FeNO, parts per billion	32.50 [20.00, 51.25]	25.5 [17.5, 38.3]	Diff −5.00	−14.00, 2.00	0.16
Pulmonary physiology					
Airway hyperresponsiveness, category					0.035
Very mild (PC_20_ ≥ 4 & <16 mg/ml, PD_20_ ≥ 0.5 & <2 mg)	39 (20)	9 (43)			
Mild (PC_20_ ≥ 1 & <4 mg/ml, PD20 ≥ 0.13 & <0.5 mg)	54 (27)	5 (24)			
Moderate (PC_20_ ≥ 0.25 & <1 mg/ml, PD_20_ ≥ 0.03 & <0.13 mg)	50 (26)	6 (28)			
Severe (PC_20_ < 0.25 mg/ml, PD20 < 0.03 mg)	54 (27)	1 (5)			
FEV_1_% of predicted normal value (pre-bronchodilator)	77.9 ± 18.9	80.0 ± 18.9	Diff +2.16	−5.93, 10.26	0.59
FEV_1_/FVC (pre-bronchodilator)	67.1 ± 11.7	64.5 ± 9.69			0.29
FEV_1_, % predicted (post-bronchodilator)	86.7 ± 17.1	90.6 ± 17.8	Diff +4.62	−2.47, 11.50	0.27
FEV_1_/FVC (post-bronchodilator)	72.2 ± 11.5	68.8 ± 9.93	Diff −3.08	−7.62, 1.41	0.15
FEV_1_ reversibility	13.8 ± 11.8	14.9 ± 13.1	Diff +1.1	−4.21, 6.48	0.66
Residual volume/total lung capacity (post-bronchodilator)	34.0 (0.10)	34.0 (8.00)	Diff 0.00	−0.04, 0.04	0.98
R5-20, kPa/L/s (post-bronchodilator)	0.06 [0.02, 0.11]	0.03 [0.00, 0.05]	Diff −0.03	−0.05, −0.01	0.0080
S_COND_, 1/L (post-bronchodilator)	0.04 [0.02, 0.06]	0.05 [0.03, 0.06]	Diff +0.01	−0.01, 0.02	0.22
S_ACIN_, 1/L (post-bronchodilator)	0.11 [0.07, 0.16]	0.15 [0.07, 0.16]	Diff +0.02	−0.02, 0.06	0.42
CT scan derived parameters					
Voxel index at-950HU	6.28 ± 6.50	4.50 ± 3.58	Diff −0.52	−3.27, 1.32	0.33
Pi10	7.15 ± 0.83	6.99 ± 1.47	Diff −0.16	−1.06, 0.73	0.54
Median lumen area, mm^2^	19.97 ± 5.47	21.3 ± 4.36	Diff +1.29	−1.48, 4.06	0.41
Median wall area, mm^2^	33.76 ± 6.12	37.3 ± 7.37	Diff +3.51	−1.04, 8.06	0.057
Median total area, mm^2^	53.96 ± 10.95	58.7 ± 11.01	Diff +4.69	−2.18, 11.55	0.15
Median lung density ratio, E/I	0.82 ± 0.08	0.84 ± 0.07	Diff +0.02	−0.03, 0.06	0.47
Lung volume ratio, E/I	0.51 ± 0.14	0.52 ± 0.13	Diff +0.01	−0.07, 0.10	0.78
Sputum-derived parameters					
Lymphocytes sputum, % of non-squamous cells	0.50 [0.20, 1.30]	0.60 [0.10, 1.60]	Diff 0.00	−0.30, 0.30	0.89
Macrophages sputum, % of non-squamous cells	32.10 [15.10, 48.00]	33.1 [18.3, 55.5]	Diff +3.00	−6.70, 13.30	0.45
Neutrophils sputum, % of non-squamous cells	53.80 [32.10, 68.30]	46.9 [21.2, 59.1]	Diff −5.60	−17.80, 5.30	0.31

Data are presented as n, n (%), mean ± SD or median [interquartile range]. Effect estimates are reported as mean differences or Hodges–Lehmann median differences for continuous variables, and as odds ratios for binary variables, with corresponding 95% confidence intervals. FeNO = fractional exhaled nitric oxide. PC_20_ = provocative concentration that causes a 20% decrease in FEV_1_ from baseline during methacholine challenge. PD_20_ = provocative dose that causes a 20% decrease in FEV_1_ from baseline during methacholine challenge. FEV_1_ = Forced expiratory volume in 1 s. FVC = forced vital capacity. Reversibility FEV_1_ (%) = percent change from initial FEV_1_ after administration of 4 × 100 μg salbutamol, calculated as: ((post-bronchodilator FEV_1_ – pre-bronchodilator FEV_1_)/pre-bronchodilator FEV_1_) × 100. RV/TLC = Residual volume/total lung capacity. R5-R20 = resistance at 5 Hz–resistance at 20 Hz. S_ACIN_ = ventilation homogeneity of the acinar zone of the lungs corrected for tidal volume. S_COND_ = ventilation heterogeneity in the conductive zone of the lungs corrected for tidal volume. Pi10 = 10 mm internal luminal perimeter.

In addition, we examined whether increasing sputum eosinophil percentages were associated with clinical and physiological characteristics (Supplementary Table S1). Higher sputum eosinophil percentages were statistically significantly associated with higher FeNO levels (β = 1.90, [95% CI 0.64, 3.17], p = 0.004). Sputum eosinophil percentages were inversely associated with FEV_1_ %predicted pre-bronchodilator (β = −1.43, [95% CI −2.47, −0.38], p = 0.008) and FEV_1_/FVC ratios both pre- (β = −1.22, [95% CI −1.85, −0.59], p = 0.0002) and post-bronchodilator (β = −1.13, [95% CI −1.72, −0.54] p = 0.0002), indicating more airflow obstruction in those with higher sputum eosinophils. Higher sputum eosinophil percentages were moreover statistically significantly associated with increased S_COND_ (β = 0.002, p = 0.047), suggesting slightly more small airways dysfunction, and a trend towards having more prior exacerbations (OR = 1.23, [95% CI 0.87, 1.25], p = 0.052).

To further explore the clinical relevance of isolated sputum eosinophilia, we performed an additional analysis adjusting for both blood eosinophilia and FeNO. A total of 98 patients had low levels of both biomarkers. Among these, 12 patients (12%) exhibited sputum eosinophilia. Clinically, these patients had a statically significantly higher GINA treatment step, more pronounced small airways dysfunction, as reflected by higher S_COND_ values, and more frequent exacerbations in the year prior to inclusion compared with those with low eosinophil levels in blood, sputum, and FeNO (Supplementary Table S2).

In addition, sensitivity analyses using alternative combinations of cut-off values to define both blood and sputum eosinophilia were performed. These analyses confirmed that even with stricter cut-off scores, a subgroup of patients remains exhibiting isolated sputum eosinophilia. Although group sizes decreased with stricter cut-offs, we observed consistent patterns with patients with isolated sputum eosinophilia continuing to show more severe airflow obstruction and small airways dysfunction. As an example, using <150 eosinophils/μL as cut-off to denote no blood eosinophilia, there was still 8% who did have sputum eosinophilia ≥2%. The full results of these analyses are presented in Supplementary Tables S3–S5.

Sex-disaggregated analyses are presented in Supplementary Tables S6 and S7. Overall, findings were generally comparable between males and females, with isolated sputum eosinophilia being associated with numerically more airflow obstruction and small airways dysfunction in both sexes, although statistical significance varied due to limited subgroup sizes.

## Discussion

In the current study, we showed that a relevant proportion of asthma patients (25/146; 17%) without elevated blood eosinophil counts still exhibit eosinophilic airway inflammation, as detected in sputum. These patients with isolated sputum eosinophilia have more severe large and small airways dysfunction, thicker airway walls on CT scans, and a higher likelihood of prior exacerbations compared to those without eosinophilia in both compartments. Of interest, the clinical characteristics of patients with isolated sputum eosinophilia were comparable to those of patients with blood eosinophilia, suggesting similar immunological mechanisms. Together, our data imply that isolated sputum eosinophilia reflects clinically relevant type 2-high inflammation in patients who would be classified as type 2-low, based on conventional biomarkers such as blood eosinophils and FeNO, suggesting that sputum eosinophilia is a more relevant biomarker as compared to blood eosinophilia.

Our study showed that asthma patients with isolated sputum eosinophilia have more severe disease expression compared to patients without eosinophilic inflammation. These patients exhibited greater impairment in both large and small airway function, showed evidence of airway remodelling as reflected by thicker airway walls on CT, and were more likely to have experienced an exacerbation in the year prior to inclusion. These structural and physiological abnormalities may be consistent with enhanced local type 2 inflammatory activity within the airways, involving mediators such as IL-13 and TGF-β, which are known to contribute to eosinophilic inflammation and airway remodelling.^18^ Our findings are partly consistent with earlier work by Schleich et al., who reported that airway eosinophilia was associated with greater airflow limitation and worse clinical outcomes.^19^ However, that study was published in 2014 and used a considerably higher threshold for blood eosinophilia (400 cells/μL) than what is used nowadays in clinical practice. We did not observe an increased risk of future exacerbations in this group. The reason for this apparent discrepancy is unclear, although it is likely related to limited statistical power. Overall, the observed physiological abnormalities suggest a potentially worse long-term prognosis if left untreated. The identification of this subgroup is therefore clinically relevant and emphasises the need for appropriate recognition and management.

The overall clinical and structural characteristics of patients with isolated sputum eosinophilia closely resembled those of patients with blood eosinophilia, supporting the concept that this subgroup represents a clinically relevant type 2-high phenotype. Interestingly, bronchial hyperresponsiveness was less pronounced in patients with isolated sputum eosinophilia compared to those with elevated blood eosinophil levels, highlighting a possible heterogeneity within type 2-high asthma.

We showed that patients with isolated sputum eosinophilia represent undetected type 2-high inflammation in individuals who would otherwise be classified as type 2-low based on conventional biomarkers such as blood eosinophils and FeNO. Notably, even when stricter thresholds for both sputum and blood eosinophilia were applied, a clinically relevant subgroup with patients demonstrating isolated sputum eosinophilia remained identifiable. Moreover, when the sputum eosinophil percentage was analysed as a continuous variable in patients with low blood eosinophils, higher sputum eosinophil levels were consistently associated with worse clinical and physiological outcomes. Thus, irrespective of the arbitrary cut-off, higher sputum eosinophil levels in patients with low blood eosinophils are of relevance. Among patients with low blood eosinophils, FeNO levels were higher in those with isolated sputum eosinophilia compared to those with low sputum eosinophils, suggesting a degree of airway eosinophilic activity may already be reflected in exhaled markers. However, importantly, even when restricting our analyses to patients with both low blood eosinophils and low FeNO, a subgroup with isolated sputum eosinophilia still remained suggesting that reliance on FeNO in combination with low blood eosinophils alone may still miss a subgroup of patients with isolated sputum eosinophilia. Previous research by Hastie et al.^14^ has shown that blood eosinophils and FeNO have limited predictive value for sputum eosinophilia. Our findings extend this by showing that patients with isolated sputum eosinophilia differ not only immunologically, but also in clinical outcomes, from those with low eosinophils in both compartments. This highlights an important gap in current practice: reliance on blood eosinophils and FeNO alone may misclassify these patients, potentially withholding opportunities for intensified anti-inflammatory therapies such as higher doses of inhaled corticosteroids or biologics targeting type 2-inflammation (3,4). Our results therefore underscore the added value of sputum analysis in comparison to conventional biomarkers.

The observed discordance between sputum and blood eosinophil levels raises important questions about the underlying mechanisms. It may reflect compartmentalised type 2 inflammation in the airways, driven by local production of cytokines such as IL-5 and IL-13. These mediators could promote the recruitment and/or retention/survival of eosinophils within the airway tissue and their migration from the circulation into the airway lumen,^20^^,^^21^ this could explain the high local, and low systemic eosinophil counts.

The prevalence of atopy was numerically higher in patients with isolated sputum eosinophilia compared to those without (91% vs. 75%). Allergic sensitisation, mediated through IgE-dependent mechanisms, is a well-established driver of type 2 airway inflammation and may induce local eosinophilic responses within the airways. However, given the limited sample size and the absence of a statistically significant difference between groups, our data do not allow firm conclusions regarding the extent to which IgE-mediated mechanisms contributed to the observed discordance between blood and sputum eosinophils.

A strength of this study is it describes a hidden eosinophilic phenotype in a comprehensively characterised adult asthma population, adding new dimensions to current phenotyping strategies. Another strength is the consistency of our findings across multiple eosinophils thresholds, which strengthens the robustness of our conclusions. However, our study also has some limitations. Data on comorbidities were not available in this cohort, therefore, the potential contribution of comorbid conditions to the observed inflammatory patterns cannot be excluded. In addition, information on medication adherence, rescue medication use, and short courses of oral corticosteroids was not available in the ATLANTIS dataset, limiting the assessment of treatment behaviour. Sputum eosinophils were measured only at baseline, and repeated assessments were not available. Consequently, we cannot assess the longitudinal stability of the isolated sputum eosinophilia phenotype. Another limitation was the small group size of patients with isolated sputum eosinophilia, possibly limiting statistical power in exacerbation outcomes. The low event rate further complicates interpretation of time-to-event analyses. In addition, higher event counts would be valuable to address recurrent event approaches, which were not addressed in this study. Residual confounding cannot be excluded, as other clinical factors that may influence exacerbation risk or airway inflammation may not have been fully captured in the dataset. Several potential sources of bias should be considered when interpreting our findings. First, sputum samples were available only in a subset of participants from selected secondary care centres, which may lead to bias of missingness and may introduce selection bias and limit the generalisability of the results. Second, exacerbations were prospectively recorded but partly relied on patient reporting between study visits, which may introduce a degree of information bias. We acknowledge that induced sputum measurements in clinical practice may be difficult to obtain, future research should therefore also focus on identifying reliable, simpler surrogate markers, such as nasal brushings or peripheral blood transcriptomic signatures. Another suggestion for future research would be to investigate whether patients with isolated sputum eosinophilia benefit from intensified therapy, including biologics, and whether this approach is cost-effective.

In conclusion, this post-hoc analysis of the ATLANTIS cohort identifies a distinct subgroup of patients with isolated sputum eosinophilia despite low blood eosinophil counts. These patients exhibited worse clinical outcomes, including more pronounced small and large airway dysfunction. Their clinical and structural characteristics were broadly comparable to those observed in patients with blood eosinophilia, suggesting that isolated sputum eosinophilia may reflect clinically relevant type 2 airway inflammation that is not captured by conventional biomarkers. These findings highlight the potential added value of direct airway inflammatory assessment in selected patients. Future prospective studies are needed to confirm these observations and to determine whether patients with isolated sputum eosinophilia may benefit from treatment strategies targeting type 2 inflammation.

## Contributors

PJMK, AMAA and LCAP contributed to data curation, formal analysis, and writing of the original draft. PJMK, AMAA and MvdB had access to and verified the data and were responsible for the decision to submit. MK, SS, LMF, BB, KFR, AP, CEB, DS, JWHK, LF, and MvdB were responsible for the conceptualisation of the study. The specific conceptualisation and design of the manuscript were carried out by MvdB. PJMK and AMAA wrote the first draft of the manuscript. HAMK, IHH, SDP, and D-JS supervised. All authors had access to the raw data, contributed to the interpretation of results, and reviewed and edited the manuscript. All authors read and approved the final version of the manuscript.

## Data sharing statement

The data underlying this analysis are available from the corresponding author (p.j.m.kuks@umcg.nl) upon reasonable request and with permission from Chiesi Farmaceutici.

## Declaration of interests

MK reports grants paid to their institution from the US National Institutes of Health, American Lung Association, Areteia, AstraZeneca, and Sanofi; personal fees for consultancy from AstraZeneca, Sanofi, Chiesi, GSK, Kinaset, and Genentech; speaker fees from Chiesi and Regeneron; travel support from the European Respiratory Society; equity in RaeSedo; leadership in the Association of Professors of Medicine; and personal fees as a Section Editor for UpToDate. SS reports speaker fees from Chiesi for presenting ATLANTIS data; travel support from the European Respiratory Society for attending science council meetings; and membership in the ATLANTIS scientific steering group. LMF reports consulting fees from Chiesi, GSK, AstraZeneca, Novartis, Verona Pharma, and Fondazione Menarini, speaker fees from Chiesi Italia, Novartis, AstraZeneca, GSK, Glenmark Pharmaceuticals limited; and participation on advisory boards for Novartis, ICON and Chiesi. BB reports honoraria for lectures from AstraZeneca, GSK, Chiesi, Sanofi and Menarini; support for attending meetings from Chiesi and AstraZeneca; and participation on advisory boards for Chiesi, GSK and AstraZeneca. KFR reports payments for lectures from AstraZeneca, Boehringer Ingelheim, Chiesi Pharmaceuticals, Novartis, Sanofi & Regeneron, GSK, Berlin Chemie, and Roche Pharma; participation on advisory boards for AstraZeneca, Boehringer Ingelheim, and Sanofi & Regeneron; and leadership roles in the German Center for Lung Research (DZL), German Chest Society (DGP), and American Thoracic Society (ATS). AP reports grants to their institution from Chiesi, AstraZeneca, GSK, and Sanofi; consulting fees from Chiesi, AstraZeneca, GSK, Novartis, Sanofi, Iqvia, Avillion, Elpen Pharmaceuticals, Moderna, and Roche; honoraria for lectures from Chiesi, AstraZeneca, GSK, Menarini, Zambon, Mundipharma, Sanofi, Edmond Pharma, Iqvia, Avillion, and Regeneron; participation on advisory boards for Chiesi, AstraZeneca, GSK, Novartis, Sanofi, Iqvia, Avillion, Elpen Pharmaceuticals, and Moderna; and receipt of materials from Consorzio Futuro in Ricerca. CEB reports grants and consultancy fees paid to their institution from 4D Pharma, Areteia, AstraZeneca, Chiesi, Genentech, GSK, Mologic, Novartis, Regeneron Pharmaceuticals, Roche, and Sanofi. DS reports consulting fees received from Adovate, Almirall, Anaveon, Apogee, Arcutis Biotherapeutics, Arrowhead, AstraZeneca, Belenos Biosciences, Bial, Celldex, Chiesi, Cipla, CONNECT Biopharm, Covis, DevPro Biopharma LCC, Elpen, Empirico, EpiEndo, Generate Biomedicines, GlaxoSmithKline, Glenmark, Jasper, Kinaset Therapeutics, KOLON, Kymera, Lupin, Melodia, Menarini, MicroA, OM Pharma, OrientEuroPharma, Recipharm, Revolo, RIGImmune Inc, Roche, Roivant Sciences, Sanofi, Sitryx, Synairgen, Tetherex, UCB, Upstream, Verona Pharma, Winward, Zura Bio, Zymeworks. AP reports being employed by Chiesi Farmaceutici. USA reports being employed by Chiesi Farmaceutici.

JWHK reports grants to their institution from AstraZeneca, Boehringer Ingelheim, Chiesi, Eli Lilly, GSK, Teva, ALK-Abello and Valneva; consulting fees from AstraZeneca, Boehringer Ingelheim, Chiesi, Eli Lilly, GSK, MSD, Novo Nordisk, Sanofi; honoraria for lectures from AstraZeneca; Boehringer Ingelheim; Chiesi; Eli Lilly; GSK; MSD; Novo Nordisk; Sanofi; Teva; ALK-Abello; Support for attending meeting and/or travel from AstraZeneca; Boehringer Ingelheim; GSK; leadership roles as IPCRG board director, CAHAG science committee; stocks as D Owner of General Practitioners Research Institute (GPRI); <5% shares of Lothar Medtec GmbH. IHH reports receiving research grants from Roche, Boehringer Ingelheim, Health Holland, Netherlands Lung Foundation, and the Dutch Research Council (NWO), outside of the submitted work. MvdB reports receiving research grants paid to their institution from GSK, Chiesi, AstraZeneca, Novartis, Genentech, and Roche. All other authors declare no competing interests. LL reports consulting fees from AstraZeneca, GSK and Sanofi; Payment for lectures for IPSA vzw and Domus Medica vzw, non-profit organisations facilitating lifelong learning for health care providers, Chiesi and Johnson and Johson.
