# Supplementary material for: Clinical phenotyping of asthma patients with elevated sputum eosinophils and low blood eosinophils: a post-hoc analysis of the multicentre ATLANTIS cohort
Source: eBioMedicine. 2026 Jul 11;130:106366. doi: 10.1016/j.ebiom.2026.106366 (PMC13382052; doi:10.1016/j.ebiom.2026.106366)
Supplement: Supplementary Material [file mmc1.docx]

**Supplementary - Clinical phenotyping of asthma in the ATLANTIS cohort: the role of sputum eosinophils in patients with low blood eosinophils – Kuks, Aabed, Premereur et al.**

Table of contents

[Supplementary table 1a: Associations between sputum eosinophil percentages (log-transformed) and clinical characteristics with binary or ordinal outcomes, in patients with low blood eosinophils (<300 cells/μL blood) 3](#_Toc232416325)

[Supplementary table 1b: Associations between sputum eosinophil percentages (log-transformed) and continous clinical characteristics in patients with low blood eosinophils (<300 cells/μL blood) 3](#_Toc232416326)

[Supplementary table 2: Baseline characteristics of asthma subjects in ATLANTIS without blood eosinophilia (<300 cells/μL blood) and without elevated nitric oxide (FeNO ≤25 part per billion) stratified for sputum eosinophilia (≥2%) 4](#_Toc232416327)

[Supplementary table 3: Baseline characteristics of asthma subjects in ATLANTIS without blood eosinophilia (<300 cells/μL blood) stratified for sputum eosinophilia (≥3%) 6](#_Toc232416328)

[Supplementary table 4: Baseline characteristics of asthma subjects in ATLANTIS without blood eosinophilia (<150 cells/μL blood) stratified for sputum eosinophilia (≥2%) 8](#_Toc232416329)

[Supplementary table 5: Baseline characteristics of asthma subjects in ATLANTIS without blood eosinophilia (<150 cells/μL blood) stratified for sputum eosinophilia (≥3%) 10](#_Toc232416330)

[Supplementary table 6: Clinical outcomes in male asthma subjects in ATLANTIS without blood eosinophilia (<300 cells/μL blood) stratified for sputum eosinophilia (≥2%) 12](#_Toc232416331)

[Supplementary table 7: Clinical outcomes in female asthma subjects in ATLANTIS without blood eosinophilia (<300 cells/μL blood) stratified for sputum eosinophilia (≥2%) 14](#_Toc232416332)

[Supplementary Table 8: Data completeness, demographics, Clinical Characteristics, Pulmonary Characteristics, CT-scan, Blood and sputum in the ATLANTIS cohort. 16](#_Toc232416333)

[ATLANTIS study protocol 18](#_Toc232416334)

| Supplementary table 1a: Associations between sputum eosinophil percentages (log-transformed) and clinical characteristics with binary or ordinal outcomes, in patients with low blood eosinophils (<300 cells/μL blood) |
| --- |

|  | OR [95% CI] | p-value |
| --- | --- | --- |
| Female sex | 0.90 [0.78, 1.03] | 0.122 |
| Smoking status | 0.96 [0.82, 1.12] | 0.605 |
| Never smoker | 1.06 [0.90, 1.24] | 0.513 |
| Ex-smoker | 0.92 [0.56, 1.50] | 0.726 |
| Current smoker | 1.03 [0.91, 1.17] | 0.593 |
| GINA classification | 1.09 [0.94, 1.27] | 0.269 |
| Use of ICS or ICS/LABA | 1.44 [0.92, 2.25] | 0.107 |
| Systemic corticosteroids use | 1.23 [1.00, 1.51] | 0.052 |
| Patients with ≥1 exacerbations in the year prior to inclusion | 1.04 [0.87, 1.25] | 0.654 |
| Positive phadiatop test | 0.96 [0.83, 1.10] | 0.532 |
| Bronchial hyperreactivity | 0.90 [0.78, 1.03] | 0.122 |
| Sputum eosinophil percentages were log-transformed to reduce skewness. OR=Odds ratio. CI=Confidence intervals. GINA=Global Initiative for Asthma (2012 guidelines). ICS=inhaled corticosteroids. ICS/LABA=/long acting beta2 agonist. | | |
| Supplementary table 1b: Associations between sputum eosinophil percentages (log-transformed) and continous clinical characteristics in patients with low blood eosinophils (<300 cells/μL blood) | | |
|  | β [95% CI] | P=value |
| Age, years | 0.416 [-0.486, 1.318] | 0.368 |
| BMI, kg/m^2^ | -0.169 [-0.567, 0.230] | 0.408 |
| Daily ICS dose (beclomethasone equivalent) in those on ICS or ICS/LABA, μg | -4.409 [-39.121, 30.303] | 0.804 |
| ACQ6 score | -0.051 [-0.125, 0.024] | 0.189 |
| Age of asthma diagnosis, years | 0.044 [-1.123, 1.212] | 0.941 |
| FeNO, parts per billion | 1.903 [0.638, 3.168] | 0.004 |
| FEV_1_ % of predicted normal value (pre-bronchodilator) | -1.427 [-2.471, -0.384] | 0.008 |
| FEV_1_/FVC (pre-bronchodilator) | -1.216 [-1.847, -0.586] | 0.000229 |
| FEV_1_, % predicted (postbronchodilator) | -0.909 [-1.834, 0.017] | 0.056 |
| FEV_1_/FVC, % (post-bronchodilator) | -1.130 [-1.719, -0.541] | 0.000249 |
| Residual volume/total lung capacity, % (post-bronchodilator) | 0.003 [-0.002, 0.009] | 0.221 |
| R5-20 (post-bronchodilator), kPa/L/s | 0.001 [-0.003, 0.006] | 0.587 |
| S_COND_, 1/L (post-bronchodilator) | 0.002 [0.000, 0.004] | 0.047 |
| S_ACIN_, 1/L (post-bronchodilator) | 0.001 [-0.006, 0.008] | 0.757 |
| Voxel index at-950HU | -0.415 [-0.853, 0.023] | 0.068 |
| Pi10 | -0.086 [-0.215, 0.044] | 0.200 |
| Median lumen area, mm^2^ | 0.062 [-0.363, 0.486] | 0.776 |
| Median airway wall area, mm^2^ | 0.474 [-0.123, 1.071] | 0.125 |
| Median totall area, mm^2^ | 0.538 [-0.401, 1.477] | 0.266 |
| Wall area/total area, % | 0.225 [-0.113, 0.562] | 0.197 |
| Median lung density ratio, E/I | 0.002 [-0.005, 0.010] | 0.537 |
| Lung volume ratio, E/I | 0.001 [-0.013, 0.015] | 0.918 |
| Sputum eosinophil percentages were log-transformed to reduce skewness. CI=Confidence intervals. BMI=Body Mass Index. ICS=inhaled corticosteroids. ICS/LABA=/long acting beta2 agonist. ACQ6=asthma control questionnaire 6. FeNO=fractional exhaled nitric oxide. FEV_1_=Forced expiratory volume in 1 second. FVC=forced vital capacity. Reversibility FEV_1_ (%)=percent RV/TLC=Residual volume/total lung capacity. R5-R20=resistance at 5 Hz–resistance at 20 Hz. S_ACIN_=ventilation homogeneity of the acinar zone of the lungs corrected for tidal volume. S_COND_=ventilation heterogeneity in the conductive zone of the lungs corrected for tidal volume. Pi10=10mm internal luminal perimeter. | | |

| Supplementary table 2: Baseline characteristics of asthma subjects in ATLANTIS without blood eosinophilia (<300 cells/μL blood) and without elevated nitric oxide (FeNO ≤25 part per billion) stratified for sputum eosinophilia (≥2%) | | | |
| --- | --- | --- | --- |
|  | **Patients without sputum eosinophilia (<2%)** | **Patients with sputum eosinophilia (≥2%)** | **p-value** |
| n | 86 | 12 |  |
| Eosinophils sputum, % of non-squamous cells | 0.10 [0.00, 0.38] | 6.15 [3.80, 10.80] | <0.001 |
| Blood eosinophil count, U/μL | 0.14 [0.09, 0.19] | 0.21 [0.17, 0.24] | 0.003 |
| **Patient characteristics** |  |  |  |
| Age, years | 43.80 (13.81) | 51.17 (12.27) | 0.083 |
| Female sex | 48 (55.8) | 5 (41.7) | 0.540 |
| BMI, kg/m^2^ | 28.28 (5.93) | 27.12 (5.16) | 0.522 |
| Smoking status |  |  |  |
| Never smoker | 70 (81.4) | 8 (66.7) | 0.422 |
| Ex-smoker | 14 (16.3) | 4 (33.3) | 0.302 |
| Current Smoker | 2 ( 2.3) | 0 ( 0.0) | 1.000 |
| Pack-years in current and ex-smokers | 3.35 [1.68, 5.00] | 5.00 [4.18, 6.00] | 0.366 |
| GINA classification |  |  | 0.032 |
| 1 | 23 (26.7) | 1 ( 8.3) |  |
| 2 | 11 (12.8) | 0 ( 0.0) |  |
| 3 | 22 (25.6) | 1 ( 8.3) |  |
| 4 | 28 (32.6) | 9 (75.0) |  |
| 5 | 2 ( 2.3) | 1 ( 8.3) |  |
| Use of ICS or ICS/LABA | 61 (70.9) | 11 (91.7) | 0.240 |
| Daily ICS dose (beclomethasone equivalent) in those on ICS or ICS/LABA, μg | 500.00 [400.00, 1000.00] | 800.00 [650.00, 800.00] | 0.502 |
| Systemic corticosteroids use | 1 (1.2) | 1 ( 8.3) | 0.578 |
| Systemic corticosteroids dose (prednisone equivalent) in those on systemic corticosteroids, mg | 5.00 [5.00, 5.00] | 20.00 [20.00, 20.00] | 0.317 |
| ACQ6 score | 0.83 [0.17, 1.67] | 0.67 [0.32, 1.04] | 0.500 |
| Age of asthma diagnosis, years | 22.00 [10.00, 36.64] | 20.85 [6.98, 43.05] | 0.970 |
| Patients with ≥1 exacerbations in the year prior to inclusion | 8 ( 9.3) | 6 (50.0) | 0.001 |
| Positive specific IgE blood screening (Phadiatop test) | 55 (75.3) | 7 (77.8) | 1.000 |
| FeNO, parts per billion | 16.00 [11.00, 19.00] | 17.00 [13.75, 22.50] | 0.191 |
| **Pulmonary physiology** |  |  |  |
| Airway hyperresponsiveness, category |  |  | 0.649 |
| Very mild | 24 (41.4) | 6 (60.0) |  |
| Mild | 18 (31.0) | 2 (20.0) |  |
| Moderate | 12 (20.7) | 1 (10.0) |  |
| Severe | 4 ( 6.9) | 1 (10.0) |  |
| FEV_1_ % of predicted normal value (pre-bronchodilator) | 86.44 (14.69) | 85.08 (20.49) | 0.776 |
| FEV_1_/FVC (pre-bronchodilator) | 71.32 (10.05) | 69.02 (7.62) | 0.449 |
| FEV_1_, % predicted (postbronchodilator) | 94.46 (12.36) | 91.22 (20.14) | 0.440 |
| FEV_1_/FVC (post-bronchodilator) | 75.67 (9.32) | 71.45 (9.16) | 0.145 |
| FEV_1_ reversibility | 11.11 (11.58) | 8.03 (6.57) | 0.372 |
| Residual volume/total lung capacity (post-bronchodilator) | 0.32 (0.07) | 0.36 (0.08) | 0.065 |
| R5-20 (post-bronchodilator), kPa/L/s | 0.04 [0.02, 0.07] | 0.04 [0.03, 0.07] | 0.731 |
| S_COND_, 1/L (post-bronchodilator) | 0.03 [0.02, 0.04] | 0.06 [0.05, 0.07] | 0.003 |
| S_ACIN_, 1/L (post-bronchodilator) | 0.10 [0.06, 0.15] | 0.16 [0.12, 0.17] | 0.259 |
| **CT scan derived parameters** | |  |  |
| Voxel index at-950HU | 5.48 (5.34) | 2.22 (1.17) | 0.121 |
| Pi10 | 7.34 (1.24) | 7.14 (1.50) | 0.705 |
| Median lumen area, mm^2^ | 20.05 (4.31) | 21.23 (5.39) | 0.535 |
| Median wall area, mm^2^ | 32.79 (5.05) | 37.39 (8.23) | 0.062 |
| Median totall area, mm2 | 53.35 (8.74) | 58.42 (12.61) | 0.209 |
| Wall area/total area, % | 62.02 (3.04) | 64.07 (4.02) | 0.138 |
| Median lung density ratio, E/I | 0.81 (0.08) | 0.87 (0.05) | 0.097 |
| Lung volume ratio, E/I | 0.52 (0.15) | 0.60 (0.13) | 0.239 |
| **Sputum-derived parameters** | |  |  |
| Lymphocytes sputum, % of non-squamous cells | 0.60 [0.30, 1.20] | 0.75 [0.08, 1.63] | 1.000 |
| Macrophages sputum, % of non-squamous cells | 41.95 [19.45, 60.90] | 32.65 [17.90, 49.42] | 0.380 |
| Neutrophils sputum, % of non-squamous cells | 51.50 [32.05, 77.88] | 51.60 [22.25, 70.38] | 0.343 |
| Data are presented as n, n (%), mean±SD or median [interquartile range]. BMI=Body Mass Index. GINA=Global Initiative for Asthma (2012 guidelines). ICS=inhaled corticosteroids. ICS/LABA=/long acting beta2 agonist. ACQ6=asthma control questionnaire 6. FeNO=fractional exhaled nitric oxide. PC_20_=provocative concentration that causes a 20% decrease in FEV_1_ from baseline during methacholine challenge. PD_20_=provocative dose that causes a 20% decrease in FEV_1_ from baseline during methacholine challenge. FEV_1_=Forced expiratory volume in 1 second. FVC=forced vital capacity. Reversibility FEV_1_ (%)=percent change from initial FEV_1_ after administration of 4×100 µg salbutamol, calculated as: ((post-bronchodilator FEV_1_ – pre-bronchodilator FEV_1_)/pre-bronchodilator FEV_1_)×100. RV/TLC=Residual volume/total lung capacity. R5-R20=resistance at 5 Hz–resistance at 20 Hz. S_ACIN_=ventilation homogeneity of the acinar zone of the lungs corrected for tidal volume. S_COND_=ventilation heterogeneity in the conductive zone of the lungs corrected for tidal volume. Pi10=10mm internal luminal perimeter. | | | |

| Supplementary table 3: Baseline characteristics of asthma subjects in ATLANTIS without blood eosinophilia (<300 cells/μL blood) stratified for sputum eosinophilia (≥3%) | | | |
| --- | --- | --- | --- |
|  | **Patients without sputum eosinophilia (<3%)** | **Patients with sputum eosinophilia (≥3%)** | **p-value** |
| n | 124 | 22 |  |
| Eosinophils sputum, % of non-squamous cells | 0.10 [0.00, 0.50] | 6.80 [4.53, 13.80] | <0.001 |
| Blood eosinophil count, U/μL | 150 [100, 200] | 190 [160, 240] | 0.017 |
| **Patient characteristics** |  |  |  |
| Age, years | 44.31 (13.47) | 47.23 (13.28) | 0.349 |
| Female sex | 72 (58.1) | 7 (31.8) | 0.041 |
| BMI, kg/m^2^ | 28.17 (6.17) | 25.89 (3.92) | 0.097 |
| Smoking status |  |  |  |
| Never smoker | 96 (77.4) | 15 (68.2) | 0.506 |
| Ex-smoker | 25 (20.2) | 7 (31.8) | 0.348 |
| Current Smoker | 3 (2.4) | 0 (0.0) | 1.000 |
| Pack-years in current and ex-smokers | 3.10 [1.60, 5.00] | 6.20 [5.00, 8.50] | 0.066 |
| GINA classification |  |  | 0.168 |
| 1 | 34 (27.4) | 3 (13.6) |  |
| 2 | 16 (12.9) | 2 (9.1) |  |
| 3 | 32 (25.8) | 5 (22.7) |  |
| 4 | 40 (32.3) | 10 (45.5) |  |
| 5 | 2 (1.6) | 2 (9.1) |  |
| Use of ICS or ICS/LABA | 87 (70.2) | 19 (86.4) | 0.190 |
| Daily ICS dose (beclomethasone equivalent) in those on ICS or ICS/LABA, μg | 700.00 [400.00, 1000.00] | 800.00 [500.00, 1000.00] | 0.518 |
| Systemic corticosteroids use | 2 (1.6) | 2 (9.1) | 0.204 |
| Systemic corticosteroids dose (prednisone equivalent) in those on systemic corticosteroids, mg | 17.50 [11.25, 23.75] | 12.50 [8.75, 16.25] | 0.683 |
| ACQ6 score | 0.82 [0.17, 1.66] | 0.67 [0.35, 1.12] | 0.515 |
| Age of asthma diagnosis, years | 22.00 [10.00, 38.05] | 28.35 [7.16, 41.87] | 0.680 |
| Patients with ≥1 exacerbations in the year prior to inclusion | 12 (9.7) | 6 (27.3) | 0.050 |
| Positive specific IgE blood screening (Phadiatop test) | 78 (75.7) | 17 (89.5) | 0.305 |
| FeNO, parts per billion | 18.00 [12.50, 26.00] | 25.00 [18.00, 42.00] | 0.011 |
| **Pulmonary physiology** |  |  |  |
| Airway hyperresponsiveness, category |  |  | 0.355 |
| Very mild | 31 (33.7) | 9 (50.0) |  |
| Mild | 35 (38.0) | 3 (16.7) |  |
| Moderate | 21 (22.8) | 5 (27.8) |  |
| Severe | 5 (5.4) | 1 (5.6) |  |
| FEV_1_ % of predicted normal value (pre-bronchodilator) | 86.76 (14.64) | 79.03 (19.93) | 0.033 |
| FEV_1_/FVC (pre-bronchodilator) | 70.80 (9.43) | 63.80 (9.70) | 0.002 |
| FEV_1_, % predicted (postbronchodilator) | 95.31 (12.76) | 89.85 (18.42) | 0.089 |
| FEV_1_/FVC (post-bronchodilator) | 75.38 (8.60) | 68.15 (10.05) | 0.001 |
| FEV_1_ reversibility | 11.36 (10.91) | 15.57 (13.66) | 0.111 |
| Residual volume/total lung capacity (post-bronchodilator) | 0.31 (0.08) | 0.35 (0.08) | 0.038 |
| R5-20 (post-bronchodilator), kPa/L/s | 0.04 [0.02, 0.08] | 0.04 [0.01, 0.06] | 0.353 |
| S_COND_, 1/L (post-bronchodilator) | 0.03 [0.02, 0.04] | 0.05 [0.03, 0.06] | 0.007 |
| S_ACIN_, 1/L (post-bronchodilator) | 0.08 [0.06, 0.15] | 0.14 [0.07, 0.16] | 0.284 |
| **CT scan derived parameters** | |  |  |
| Voxel index at-950HU | 5.22 (4.78) | 4.50 (3.58) | 0.617 |
| Pi10 | 7.37 (1.28) | 6.99 (1.47) | 0.358 |
| Median lumen area, mm^2^ | 20.00 (4.25) | 21.26 (4.36) | 0.348 |
| Median wall area, mm^2^ | 32.88 (5.45) | 37.27 (7.37) | 0.021 |
| Median totall area, mm2 | 53.59 (8.92) | 58.65 (11.01) | 0.091 |
| Wall area/total area, % | 62.00 (3.44) | 63.69 (3.23) | 0.120 |
| Median lung density ratio, E/I | 0.81 (0.08) | 0.84 (0.07) | 0.275 |
| Lung volume ratio, E/I | 0.50 (0.14) | 0.52 (0.13) | 0.574 |
| **Sputum-derived parameters** | |  |  |
| Lymphocytes sputum, % of non-squamous cells | 0.65 [0.30, 1.52] | 0.40 [0.10, 0.90] | 0.248 |
| Macrophages sputum, % of non-squamous cells | 43.70 [19.58, 63.85] | 33.50 [20.25, 54.40] | 0.429 |
| Neutrophils sputum, % of non-squamous cells | 50.50 [28.17, 76.15] | 46.85 [21.55, 55.98] | 0.135 |
| Data are presented as n, n (%), mean±SD or median [interquartile range]. BMI=Body Mass Index. GINA=Global Initiative for Asthma (2012 guidelines). ICS=inhaled corticosteroids. ICS/LABA=/long acting beta2 agonist. ACQ6=asthma control questionnaire 6. FeNO=fractional exhaled nitric oxide. PC_20_=provocative concentration that causes a 20% decrease in FEV_1_ from baseline during methacholine challenge. PD_20_=provocative dose that causes a 20% decrease in FEV_1_ from baseline during methacholine challenge. FEV_1_=Forced expiratory volume in 1 second. FVC=forced vital capacity. Reversibility FEV_1_ (%)=percent change from initial FEV_1_ after administration of 4×100 µg salbutamol, calculated as: ((post-bronchodilator FEV_1_ – pre-bronchodilator FEV_1_)/pre-bronchodilator FEV_1_)×100. RV/TLC=Residual volume/total lung capacity. R5-R20=resistance at 5 Hz–resistance at 20 Hz. S_ACIN_=ventilation homogeneity of the acinar zone of the lungs corrected for tidal volume. S_COND_=ventilation heterogeneity in the conductive zone of the lungs corrected for tidal volume. Pi10=10mm internal luminal perimeter. | | | |

| Supplementary table 4: Baseline characteristics of asthma subjects in ATLANTIS without blood eosinophilia (<150 cells/μL blood) stratified for sputum eosinophilia (≥2%) | | | |
| --- | --- | --- | --- |
|  | **Patients without sputum eosinophilia (<2%)** | **Patients with sputum eosinophilia (≥2%)** | **p-value** |
| n | 58 | 5 |  |
| Eosinophils sputum, % of non-squamous cells | 0.10 [0.00, 0.30] | 5.80 [4.10, 6.90] |  |
| Blood eosinophil count, U/μL | 100 [80, 120] | 100 [80, 140] | 0.789 |
| **Patient characteristics** |  |  |  |
| Age, years | 46.93 (13.16) | 51.80 (4.97) | 0.417 |
| Female sex | 40 (69.0) | 2 (40.0) | 0.410 |
| BMI, kg/m^2^ | 26.67 (5.49) | 23.31 (4.42) | 0.190 |
| Smoking status |  |  |  |
| Never smoker | 44 (75.9) | 2 (40.0) | 0.227 |
| Ex-smoker | 12 (20.7) | 3 (60.0) | 0.152 |
| Current Smoker | 2 (3.4) | 0 (0.0) | 1.000 |
| Pack-years in current and ex-smokers | 3.10 [1.23, 5.00] | 6.20 [5.60, 7.60] | 0.146 |
| GINA classification |  |  | 0.510 |
| 1 | 17 (29.3) | 0 (0.0) |  |
| 2 | 4 (6.9) | 1 (20.0) |  |
| 3 | 15 (25.9) | 1 (20.0) |  |
| 4 | 21 (36.2) | 3 (60.0) |  |
| 5 | 1 (1.7) | 0 (0.0) |  |
| Use of ICS or ICS/LABA | 41 (70.7) | 5 (100.0) | 0.373 |
| Daily ICS dose (beclomethasone equivalent) in those on ICS or ICS/LABA, μg | 800.00 [500.00, 1000.00] | 800.00 [500.00, 1000.00] | 0.836 |
| Systemic corticosteroids use | 0 (0.0) | 0 (0.0) | NaN |
| Systemic corticosteroids dose (prednisone equivalent) in those on systemic corticosteroids, mg | NA [NA, NA] | NA [NA, NA] | NA |
| ACQ6 score | 0.82 [0.17, 1.78] | 0.50 [0.00, 1.16] | 0.349 |
| Age of asthma diagnosis, years | 24.79 [10.04, 41.68] | 39.00 [28.70, 41.26] | 0.321 |
| Patients with ≥1 exacerbations in the year prior to inclusion | 6 (10.3) | 0 (0.0) | 1.000 |
| Positive specific IgE blood screening (Phadiatop test) | 35 (74.5) | 4 (80.0) | 1.000 |
| FeNO, parts per billion | 17.00 [12.00, 22.50] | 30.00 [28.00, 37.00] | 0.004 |
| **Pulmonary physiology** |  |  |  |
| Airway hyperresponsiveness, category |  |  | 0.418 |
| Very mild | 18 (37.5) | 0 (0.0) |  |
| Mild | 13 (27.1) | 2 (66.7) |  |
| Moderate | 14 (29.2) | 1 (33.3) |  |
| Severe | 3 (6.2) | 0 (0.0) |  |
| FEV_1_ % of predicted normal value (pre-bronchodilator) | 86.84 (16.06) | 74.66 (24.85) | 0.124 |
| FEV_1_/FVC (pre-bronchodilator) | 70.62 (10.00) | 55.59 (10.50) | 0.002 |
| FEV_1_, % predicted (postbronchodilator) | 96.44 (12.06) | 85.65 (25.52) | 0.090 |
| FEV_1_/FVC (post-bronchodilator) | 75.89 (8.56) | 59.86 (12.83) | <0.001 |
| FEV_1_ reversibility | 13.80 (13.84) | 15.97 (8.43) | 0.733 |
| Residual volume/total lung capacity (post-bronchodilator) | 0.32 (0.07) | 0.39 (0.07) | 0.038 |
| R5-20 (post-bronchodilator), kPa/L/s | 0.03 [0.01, 0.06] | 0.02 [0.00, 0.03] | 0.402 |
| S_COND_, 1/L (post-bronchodilator) | 0.03 [0.01, 0.04] | 0.03 [0.02, 0.03] | 0.949 |
| S_ACIN_, 1/L (post-bronchodilator) | 0.08 [0.06, 0.14] | 0.15 [0.11, 0.19] | 0.656 |
| **Sputum-derived parameters** | |  |  |
| Lymphocytes sputum, % of non-squamous cells | 0.65 [0.20, 1.30] | 0.30 [0.00, 0.60] | 0.108 |
| Macrophages sputum, % of non-squamous cells | 34.60 [20.10, 59.30] | 38.70 [18.30, 47.40] | 0.576 |
| Neutrophils sputum, % of non-squamous cells | 53.20 [34.90, 75.62] | 46.90 [36.30, 54.30] | 0.647 |
| Data are presented as n, n (%), mean±SD or median [interquartile range]. BMI=Body Mass Index. GINA=Global Initiative for Asthma (2012 guidelines). ICS=inhaled corticosteroids. ICS/LABA=/long acting beta2 agonist. ACQ6=asthma control questionnaire 6. FeNO=fractional exhaled nitric oxide. PC_20_=provocative concentration that causes a 20% decrease in FEV_1_ from baseline during methacholine challenge. PD_20_=provocative dose that causes a 20% decrease in FEV_1_ from baseline during methacholine challenge. FEV_1_=Forced expiratory volume in 1 second. FVC=forced vital capacity. Reversibility FEV_1_ (%)=percent change from initial FEV_1_ after administration of 4×100 µg salbutamol, calculated as: ((post-bronchodilator FEV_1_ – pre-bronchodilator FEV_1_)/pre-bronchodilator FEV_1_)×100. RV/TLC=Residual volume/total lung capacity. R5-R20=resistance at 5 Hz–resistance at 20 Hz. S_ACIN_=ventilation homogeneity of the acinar zone of the lungs corrected for tidal volume. S_COND_=ventilation heterogeneity in the conductive zone of the lungs corrected for tidal volume. Pi10=10mm internal luminal perimeter. | | | |

| Supplementary table 5: Baseline characteristics of asthma subjects in ATLANTIS without blood eosinophilia (<150 cells/μL blood) stratified for sputum eosinophilia (≥3%) | | | |
| --- | --- | --- | --- |
|  | **Patients without sputum eosinophilia (<3%)** | **Patients with sputum eosinophilia (≥3%)** | **p-value** |
| n | 59 | 4 |  |
| Eosinophils sputum, % of non-squamous cells | 0.10 [0.00, 0.30] | 6.35 [5.38, 13.58] |  |
| Blood eosinophil count, U/μL | 10 [80, 120] | 90 [60, 110] | 0.621 |
| **Patient characteristics** |  |  |  |
| Age, years | 47.10 (13.11) | 50.50 (4.65) | 0.610 |
| Female sex | 41 (69.5) | 1 (25.0) | 0.201 |
| BMI, kg/m^2^ | 26.58 (5.48) | 23.73 (4.99) | 0.315 |
| Smoking status |  |  |  |
| Never smoker | 44 (74.6) | 2 (50.0) | 0.624 |
| Ex-smoker | 13 (22.0) | 2 (50.0) | 0.506 |
| Current Smoker | 2 (3.4) | 0 (0.0) | 1.000 |
| Pack-years in current and ex-smokers | 3.50 [1.45, 7.00] | 5.60 [5.30, 5.90] | 0.369 |
| GINA classification |  |  | 0.249 |
| 1 | 17 (28.8) | 0 (0.0) |  |
| 2 | 4 (6.8) | 1 (25.0) |  |
| 3 | 16 (27.1) | 0 (0.0) |  |
| 4 | 21 (35.6) | 3 (75.0) |  |
| 5 | 1 (1.7) | 0 (0.0) |  |
| Use of ICS or ICS/LABA | 42 (71.2) | 4 (100.0) | 0.500 |
| Daily ICS dose (beclomethasone equivalent) in those on ICS or ICS/LABA, μg | 800.00 [500.00, 1000.00] | 900.00 [725.00, 1250.00] | 0.361 |
| Systemic corticosteroids use | 0 (0.0) | 0 (0.0) | NaN |
| Systemic corticosteroids dose (prednisone equivalent) in those on systemic corticosteroids, mg | NA [NA, NA] | NA [NA, NA] | NA |
| ACQ6 score | 0.83 [0.17, 1.75] | 0.25 [0.00, 0.79] | 0.232 |
| Age of asthma diagnosis, years | 25.60 [10.07, 41.36] | 34.98 [24.15, 43.49] | 0.430 |
| Patients with ≥1 exacerbations in the year prior to inclusion | 6 (10.2) | 0 (0.0) | 1.000 |
| Positive specific IgE blood screening (Phadiatop test) | 36 (75.0) | 3 (75.0) | 1.000 |
| FeNO, parts per billion | 17.50 [12.00, 23.50] | 33.50 [28.75, 38.25] | 0.009 |
| **Pulmonary physiology** |  |  |  |
| Airway hyperresponsiveness, category |  |  | 0.172 |
| Very mild | 18 (36.7) | 0 (0.0) |  |
| Mild | 13 (26.5) | 2 (100.0) |  |
| Moderate | 15 (30.6) | 0 (0.0) |  |
| Severe | 3 (6.1) | 0 (0.0) |  |
| FEV_1_ % of predicted normal value (pre-bronchodilator) | 86.93 (15.94) | 70.35 (26.46) | 0.058 |
| FEV_1_/FVC (pre-bronchodilator) | 70.42 (10.02) | 54.63 (11.87) | 0.004 |
| FEV_1_, % predicted (postbronchodilator) | 96.66 (12.07) | 79.75 (25.23) | 0.015 |
| FEV_1_/FVC (post-bronchodilator) | 75.67 (8.65) | 59.05 (14.67) | 0.001 |
| FEV_1_ reversibility | 13.89 (13.74) | 15.23 (9.54) | 0.849 |
| Residual volume/total lung capacity (post-bronchodilator) | 0.32 (0.07) | 0.41 (0.07) | 0.015 |
| R5-20 (post-bronchodilator), kPa/L/s | 0.03 [0.01, 0.06] | 0.01 [0.00, 0.03] | 0.513 |
| S_COND_, 1/L (post-bronchodilator) | 0.03 [0.01, 0.04] | 0.04 [0.04, 0.04] | 0.328 |
| S_ACIN_, 1/L (post-bronchodilator) | 0.08 [0.06, 0.14] | 0.06 [0.06, 0.06] | 0.477 |
| **Sputum-derived parameters** | |  |  |
| Lymphocytes sputum, % of non-squamous cells | 0.60 [0.20, 1.30] | 0.15 [0.00, 0.40] | 0.080 |
| Macrophages sputum, % of non-squamous cells | 34.30 [19.45, 59.20] | 43.05 [33.60, 48.32] | 0.978 |
| Neutrophils sputum, % of non-squamous cells | 54.60 [35.50, 76.70] | 41.60 [33.73, 48.75] | 0.260 |
| Data are presented as n, n (%), mean±SD or median [interquartile range]. BMI=Body Mass Index. GINA=Global Initiative for Asthma (2012 guidelines). ICS=inhaled corticosteroids. ICS/LABA=/long acting beta2 agonist. ACQ6=asthma control questionnaire 6. FeNO=fractional exhaled nitric oxide. PC_20_=provocative concentration that causes a 20% decrease in FEV_1_ from baseline during methacholine challenge. PD_20_=provocative dose that causes a 20% decrease in FEV_1_ from baseline during methacholine challenge. FEV_1_=Forced expiratory volume in 1 second. FVC=forced vital capacity. Reversibility FEV_1_ (%)=percent change from initial FEV_1_ after administration of 4×100 µg salbutamol, calculated as: ((post-bronchodilator FEV_1_ – pre-bronchodilator FEV_1_)/pre-bronchodilator FEV_1_)×100. RV/TLC=Residual volume/total lung capacity. R5-R20=resistance at 5 Hz–resistance at 20 Hz. S_ACIN_=ventilation homogeneity of the acinar zone of the lungs corrected for tidal volume. S_COND_=ventilation heterogeneity in the conductive zone of the lungs corrected for tidal volume. Pi10=10mm internal luminal perimeter. | | | |

| Supplementary table 6: Clinical outcomes in male asthma subjects in ATLANTIS without blood eosinophilia (<300 cells/μL blood) stratified for sputum eosinophilia (≥2%) | | | |
| --- | --- | --- | --- |
|  | **Patients without sputum eosinophilia (<2%)** | **Patients with sputum eosinophilia (≥2%)** | **p-value** |
| n | 52 | 15 |  |
| Eosinophils sputum, % of non-squamous cells | 0.10 [0.00, 0.30] | 9.80 [4.55, 19.40] | <0.001 |
| Patients with ≥1 exacerbations in the year prior to inclusion, n (%) | 2 ( 3.8) | 3 (20.0) | 0.124 |
| Positive specific IgE blood screening (Phadiatop test) | 38 (73.1) | 12 (80.0) | 0.682 |
| FeNO, parts per billion | 19.00 [15.50, 25.50] | 25.50 [19.00, 39.50] | 0.061 |
| **Pulmonary physiology** |  |  |  |
| FEV_1_ % of predicted normal value (pre-bronchodilator) | 85.02 (13.34) | 75.79 (20.93) | 0.044 |
| FEV_1_/FVC (pre-bronchodilator) | 0.69 (0.10) | 0.63 (0.11) | 0.028 |
| FEV_1_, % predicted (post-bronchodilator) | 94.47 [88.66, 103.89] | 89.17 [77.78, 100.50] | 0.191 |
| FEV_1_/FVC, % (post-bronchodilator) | 0.74 (0.09) | 0.67 (0.11) | 0.012 |
| Residual volume/total lung capacity, % (post-bronchodilator) | 28.0 (7.0) | 33.0 (8.0) | 0.020 |
| R5-20 (post-bronchodilator), kPa/L/s | 0.04 (0.06) | 0.06 (0.08) | 0.565 |
| S_COND_, 1/L (post-bronchodilator) | 0.03 [0.02, 0.04] | 0.05 [0.03, 0.06] | 0.051 |
| **CT scan derived parameters** | |  |  |
| Pi10 | 7.47 (1.32) | 7.03 (1.78) | 0.487 |
| Voxel index at-950HU | 5.52 [2.06, 8.29] | 4.26 [2.15, 8.57] | 0.865 |
| Median lumen area, mm^2^ | 21.60 (4.64) | 21.17 (4.57) | 0.823 |
| Median airway wall area, mm^2^ | 34.94 (5.62) | 38.01 (8.25) | 0.279 |
| Median totall area, mm^2^ | 57.29 (9.39) | 59.34 (12.14) | 0.641 |
| Wall area/total area, % | 61.68 (4.02) | 64.23 (3.27) | 0.119 |
| Median lung density ratio, E/I | 0.82 (0.07) | 0.82 (0.07) | 0.812 |
| Lung volume ratio, E/I | 0.51 (0.13) | 0.49 (0.12) | 0.670 |

| Supplementary table 7: Clinical outcomes in female asthma subjects in ATLANTIS without blood eosinophilia (<300 cells/μL blood) stratified for sputum eosinophilia (≥2%) | | | |
| --- | --- | --- | --- |
|  | **Patients without sputum eosinophilia (<2%)** | **Patients with sputum eosinophilia (≥2%)** | **p-value** |
| n | 69 | 10 |  |
| Eosinophils sputum, % of non-squamous cells | 0.10 [0.00, 0.50] | 5.20 [2.62, 6.85] | <0.001 |
| Patients with ≥1 exacerbations in the year prior to inclusion, n (%) | 9 (13.0) | 4 (40.0) | 0.091 |
| Positive specific IgE blood screening (Phadiatop test) | 38 (55.1) | 7 (70.0) | 0.559 |
| FeNO, parts per billion | 17.00 [11.00, 26.00] | 26.00 [15.75, 35.00] | 0.072 |
| **Pulmonary physiology** |  |  |  |
| FEV_1_ % of predicted normal value (pre-bronchodilator) | 88.05 (15.78) | 86.37 (14.12) | 0.751 |
| FEV_1_/FVC (pre-bronchodilator) | 0.72 (0.09) | 0.68 (0.07) | 0.136 |
| FEV_1_, % predicted (post-bronchodilator) | 97.53 [88.48, 105.48] | 99.90 [89.26, 105.12] | 0.892 |
| FEV_1_/FVC, % (post-bronchodilator) | 0.77 (0.08) | 0.72 (0.07) | 0.096 |
| Residual volume/total lung capacity, % (post-bronchodilator) | 0.33 (0.07) | 0.35 (0.10) | 0.523 |
| R5-20 (post-bronchodilator), kPa/L/s | 0.06 (0.07) | 0.02 (0.03) | 0.041 |
| S_COND_, 1/L (post-bronchodilator) | 0.03 [0.02, 0.05] | 0.05 [0.04, 0.06] | 0.148 |
| S_ACIN_, 1/L (post-bronchodilator) | 0.10 [0.06, 0.14] | 0.16 [0.11, 0.18] | 0.093 |
|  | |  |  |
| Pi10 | 7.32 (1.28) | 6.90 (0.48) | 0.518 |
| Voxel index at-950HU | 3.24 [2.47, 5.71] | 2.33 [1.94, 2.50] | 0.087 |
| Median lumen area, mm^2^ | 19.16 (3.86) | 21.48 (4.52) | 0.275 |
| Median airway wall area, mm^2^ | 31.81 (5.13) | 35.60 (5.51) | 0.177 |
| Median totall area, mm^2^ | 51.69 (8.18) | 57.08 (9.37) | 0.230 |
| Wall area/total area, % | 62.17 (3.17) | 62.46 (3.21) | 0.865 |
| Median lung density ratio, E/I | 0.80 (0.08) | 0.88 (0.04) | 0.083 |
| Lung volume ratio, E/I | 0.49 (0.15) | 0.60 (0.13) | 0.189 |

| Supplementary Table 8: Data completeness, demographics, Clinical Characteristics, Pulmonary Characteristics, CT-scan, Blood and sputum in the ATLANTIS cohort. | | |
| --- | --- | --- |
|  |  | **Data available, n** |
| Patients | 773 |  |
| **Demographics and clinical characteristics** |  |  |
| Female sex | 450 (58%) | 773 |
| Age at inclusion, years | 44.3±12.99 | 773 |
| BMI, kg/m^2^ | 27.23±5.88 | 773 |
| Smoking status |  | 767 |
| - Never smoker | 585 (76%) |  |
| - Ex-smoker | 154 (20%) |  |
| - Current smoker | 28 (4%) |  |
| Pack-years (in all subjects) | 4.34 [2.00, 7.50] | 767 |
| GINA treatment step  1  2  3  4  5 | 135 (17%)  85 (11%)  207 (27%)  300 (39%)  46 (6%) | 773 |
| Age of asthma diagnosis, years | 24.05 [9.35, 40.89] | 769 |
| Exacerbations during follow-up | 0.00 [0.00, 0.00] | 752 |
| Positive phadiatop test | 454 (80.5%) | 564 |
| Airway hyperresponsiveness category |  | 556 |
| - Very mild | 141 (25.4%) |  |
| - Mild | 169 (30.4%) |  |
| - Moderate | 134 (24.1%) |  |
| - Severe | 112 (20.1%) |  |
| FeNO. Parts per billion | 25.00 [16.00, 38.00] | 627 |
| ACQ6 score | 0.80 [0.30, 1.50] | 772 |
| **Pulmonary traits** |  |  |
| FEV_1,_ % predicted (post-bronchodilator) | 89.3±16.0 | 760 |
| FEF_25-75_, % predicted (post-bronchodilator) | 76.8±36.10 | 720 |
| RV/TLC, % | 33.1±9.05 | 693 |
| R_5-20_, kPa/L/s | 0.05 [0.02, 0.09] | 617 |
| S_COND_ ,1/L | 0.03 [0.02, 0.04] | 380 |
| **CT-scan-derived parameters** |  |  |
| Wall area/total area % | 63.03±3.47 | 304 |
| VI 950% | 3.64 [1.46, 7.75] | 304 |
| Mean lung density ratio, E/I | 0.83 [0.77, 0.88] | 292 |
| Lung volume ratio, E/I | 0.83 [0.77, 0.88] | 292 |
| **Blood-derived parameters** |  | 767 |
| Blood basophil count, 10^9^/L | 0.03 [0.02, 0.06] |  |
| Blood eosinophil count, 10^9^/L | 0.23 [0.13, 0.38] |  |
| Blood lymphocyte count, 10^9^/L | 1.87 [1.57, 2.26] |  |
| Blood monocyte count, 10^9^/L | 0.46 [0.38, 0.58] |  |
| Blood neutrophil count, 10^9^/L | 3.68 [2.96, 4.70] |  |
| **Sputum-derived parameters** |  | 228 |
| Bronchial epithelial cells sputum, % of non-squamous cells | 1.80 [0.80, 4.03] |  |
| Eosinophils sputum, % of non-squamous cells | 0.50 [0.10, 2.82] |  |
| Lymphocytes sputum, % of non-squamous cells | 0.60 [0.30, 1.33] |  |
| Macrophages sputum, % of non-squamous cells | 36.40 [18.45, 59.50] |  |
| Neutrophils sputum, % of non-squamous cells | 50.80 [26.42, 70.62] |  |
| Data are presented as n, n (%), mean±SD or median [interquartile range]. BMI=Body Mass Index. GINA treatment steps were determined based on medication usage, according to the 2012 guidelines. Very mild airway hyperresponsiveness = PC_20_ ≥ 4 & < 16 mg/mL, PD_20_ ≥ 0.5 & < 2 mg. Mild airway hyperresponsiveness = PC_20_ ≥ 1 & < 4 mg/mL, PD20 ≥ 0.13 & < 0.5 mg. Moderate airway hyperresponsiveness = PC_20_ ≥ 0.25 & <1 mg/mL, PD_20_ ≥ 0.03 & < 0.13 mg. Severe airway hyperresponsiveness = PC_20_ < 0.25 mg/mL, PD20 <0.03 mg. FeNO=fractional exhaled nitric oxide. ACQ6=asthma control questionnaire 6. FEV_1_=Forced expiratory volume in 1 second. RV/TLC=Residual volume/total lung capacity. S_cond_=ventilation heterogeneity in the conductive zone of the lungs corrected for tidal volume. R_5-20_=resistance at 5 Hz–resistance at 20 Hz. VI 950=Voxel index at -950 Hounsfield units. E/I=expiratory/inspiratory. | | |

# ATLANTIS study protocol

| **Study title** | **A**ssessen**T** of smal**L A**irways involveme**NT I**n a**S**thma (**ATLANTIS**) |
| --- | --- |
| **Sponsor** | Chiesi Farmaceutici S.p.A. - Via Palermo 26/A, 43122 Parma - Italy |
| **Centre(s)** | ~31 (Italy, Germany, Canada, UK, The Netherlands, USA, China, Brazil and Spain) |
| **Indication** | Bronchial asthma |
| **Study design** | Multinational, multicentre, non-pharmacological intervention, cross-sectional and longitudinal study. |
| **Major objectives** | - To determine the role of small airways abnormalities in the clinical manifestations of asthma. - To evaluate which (combination of) clinical methods best assess the abnormalities of small airways and large airways disease in asthma and best relates to asthma severity, control, and future risk of exacerbations, both cross-sectionally and longitudinally. - To assess if a q uestionnaire (**S**mall **A**irways **D**ysfunction **T**ool) could be offered to physicians in diagnosing Small Airways Disease (SAD) in asthma, and thus   characterize asthma patients with small airways diseases as determined by physiologic and radiographic assessments and measurement of specific biomarkers. |
| **Minor objectives** | - To define the physiologic characteristics that correlate with small airways function in asthma as compared to healthy controls. - To define the radiographic characteristics that correlate with small airways function in asthma as compared to healthy controls. - To determine which direct and indirect measures of inflammation best correlate with inflammation in the large and small airway compartments. - To determine if questionnaires such as ACQ-6 and ACT assess small airways function. - To determine the correlation between SAD and asthma control. - To determine if SAD is associated with exacerbations requiring prescription of oral corticosteroids. |
| **Study duration** | 12 months |
| **Number of subjects** | 900 subjects divided into 800 asthmatic patients and 100 healthy controls will be included. Recruitment will be balanced, at the extent possible, in order to achieve at least > 150 patients each for steps 1 - 4 and > 50 patients for step 5 as indicated in the international guidelines (GINA 2012) on the basis of their previous therapy. The 100 healthy controls  will be recruited on the basis of absence of respiratory symptoms, airway obstruction and hyper responsiveness. |
| **Inclusion/exclusion criteria** | **Asthmatic patient inclusion criteria**   1. Male or female patients aged ≥ 18 and ≤ 65 years, who have signed an Informed Consent form prior to initiation of any study-related procedure. 2. Clinical diagnosis of asthma for at least 6 months confirmed by a chest physician according to international guidelines (GINA 2012) supported by objective evidence of any of the following at the baseline visit or in the previous 5 years.    1. Positive response to methacholine challenge test [PC_20_ < 8 mg/mL or PD_20_ < 0.7 mg for those subjects not using inhaled corticosteroids (ICS), and PC_20_ < 16 mg/mL or PD_20_ < 1.4 mg for subjects using ICS]   ***or***   - 1. Positive response to a reversibility test, defined as ΔFEV_1_ ≥ 12% and ≥ 200 mL over baseline FEV_1_, within 30 minutes after administration of   400 μg of salbutamol pMDI administered with or without Spacer |

|  | ***or***   1. Peak Flow variability (i.e. highest - lowest PEF over the day/mean value of the two, × 100) > 20%, measured over a follow-up period of 7 days   ***or***   1. Documented response (defined as ΔFEV_1_ ≥ 12% and ≥ 200 mL) after a cycle (e.g., 4 weeks) of regular maintenance anti-asthma treatment. 2. Patients with stable asthma, on any previous regular asthma treatment (“rescue” β_2_-agonists alone included) at a stable dose, for at least 8 weeks prior to baseline visit. 3. Current smoker, ex-smoker (since the past 12 months) or lifelong non-smoker (total lifetime smoking history < 10 packyears defined as [(number of cigarettes smoked per day)x(number of years of smoking)] / 20).   **Healthy subject inclusion criteria**   1. Male or female patients aged ≥ 18 and ≤ 65 years, who have signed the Informed Consent form prior to initiation of any study-related procedure. 2. No clinical history of asthma or COPD (no respiratory symptoms compatible to asthma or COPD in the past 2 years). 3. Current smoker, ex-smoker (since the past 12 months) or lifelong non-smoker (total lifetime smoking history < 10 packyears). 4. Normal spirometry: baseline FEV1 ≥ 80% of the predicted normal value, FEV1/FVC > LLN (lower limit of normal). 5. Normal airways responsiveness: PC20 ≥ 16 mg/mL, PD20 ≥ 1.4 mg.   **Asthmatic patient exclusion criteria**   1. Cigarette smoking > 10 packyears defined as [(number of cigarettes smoked per day) x (number of years of smoking)] / 20. 2. Diagnosis of COPD confirmed by a chest physician. 3. Asthma exacerbation in the 8 weeks prior to baseline visit (defined as a significant deterioration of asthma and signalled by any or more of the following: need for a systemic corticosteroid course (≥ 3 days); hospitalisation for asthma; emergency room attendance for asthma). 4. Clinical or functional uncontrolled respiratory, haematological, immunologic, renal, neurologic, hepatic, endocrinal or other disease, or any condition that might, in the judgment of the investigator, compromise the results or interpretation of the study. 5. Pregnant or lactating women (a urine pregnancy test will be performed). 6. Participation in an interventional clinical trial with intake of the last dose of any investigational drug <12 weeks preceding baseline visit (last dose < 5 half-lives prior to baseline visit for biologics). 7. Inability to comply with study procedures. 8. Alcohol or drug abuse.   **Healthy subject exclusion criteria**   1. Cigarette smoking history > 10 packyears defined as [(number of cigarettes smoked per day) x (number of years of smoking)] / 20. 2. Diagnosed upper and/or lower respiratory disease(s). 3. Clinical or functional uncontrolled haematological, immunologic, renal, neurologic, hepatic, endocrinal or other disease, or any condition that might, in the judgment of the investigator, compromise the results or interpretation of the study. 4. Pregnant or lactating women (a urine pregnancy test will be performed). 5. Participation in an interventional clinical trial with intake of the last dose of any investigational drug <12 weeks preceding baseline visit (last dose < 5 half-lives |
| --- | --- |

|  | prior to baseline visit for biologics).   1. Inability to comply with study procedures. 2. Alcohol or drug abuse. |
| --- | --- |
| **Study plan** | Throughout the study, various assessments and tests will be performed according to the Study Flow Diagram ([Table 1](#_bookmark10)).  A total of three clinic visits (the first divided into 2 or 3 days) and two phone contacts will be performed during the study:   - Visit 1 (**V1**): baseline visit, cross-sectional phase, start of 12-month longitudinal period. - Visit 2 (**V2**): follow-up after 6 months from baseline visit. - Visit 3 (**V3**): follow-up after 12 months from baseline visit. - *Follow-up phone contacts*: after 3 and 9 months from baseline visits. |
| **Investigations** | - Demographics: age, gender, height, weight, vital signs - Asthma exacerbations (defined as a significant deterioration of asthma and signalled by any or more of the following: need for a systemic corticosteroid course (≥ 3 days); hospitalisation for asthma; emergency room attendance for asthma).   **Questionnaires:**   - Asthma Control Test (ACT); - Asthma Control Questionnaire (ACQ-6); - Bronchial Hyper responsiveness Questionnaire (BHQ) – selected sites only; - Mini Asthma Quality of Life Questionnaire (mini-AQLQ); - Standardised measure of health status descriptive system (EuroQol-5D-5L); - Small Airways Dysfunction Tool (SADT); - Morisky Medication Adherence Scale (MMAS-8).   **Measurements of Lung Physiology:**   - Multiple-Breath Nitrogen Washout (MBNW); - Impulse oscillometry (IOS) - where applicable, depending on the availability of the equipment at the site; - Spirometry; - Body box; - Methacholine challenge test.   **Measurement of inflammation and biomarkers:**   - Blood total and differential cell count; - Phadiatop; - Optional serum, plasma and blood cells collection will be applicable only in EU, US (selected sites only) and Canada sites. For those subjects who consented for the optional collection of serum, plasma and blood cells, samples will be stored for future analysis (including genetic tests on DNA) on the pathobiology of asthma (asthma-related biomarkers) which will be defined in future specific protocols; - Sputum induction: differential cell count -selected sites in EU, US (selected sites only) and Canada. Optional collection of sputum supernatant will be also applicable. For those subjects who consented for the optional collection, sputum supernatant will be stored for future analysis on the pathobiology of asthma which will be defined in future specific protocols; - Nasal brushing: for those subjects consenting for this optional assessment in all sites in EU, US (selected sites only) and Canada, RNA will be extracted and stored, and cells will be cryopreserved for future analysis on the pathobiology of asthma which will be defined in future specific protocols; - Fraction of exhaled NO (FeNO) – where applicable, depending on the availability of the equipment at the site; - Optional bronchoscopy with endobronchial and trans-bronchial biopsy will be |

|  | applicable in selected sites only. Total number of inflammatory cells and quantification of specific cells including eosinophils, neutrophils, T cells and macrophages will be performed. Gene expression profiles (including RNA) will be performed at a later date for future analysis on the pathobiology of asthma which will be defined in future specific protocols.  **Imaging:**  - Thoracic Computed Tomography (CT) scan in a subgroup of ~ 530 asthmatic patients balanced, at the extent possible, into the five treatment groups (see section 4.1 “Subject recruitment”) as indicated in the international guidelines (GINA) and 50 healthy controls (580 subjects in total). CT scan will be conducted in selected sites only |
| --- | --- |
| **Health resource consumption variables** | - Asthma-specific hospital admissions (number and length); - Asthma-specific emergency room or urgent care visits; - Unscheduled consultations for asthma (without hospitalisation) defined as: the need for a visit (Medical Specialist or General Practitioner) due to symptoms worsening; - Unscheduled tests for asthma (without hospitalisation). |
| **Sample size calculation** | 800 patients will be sufficient to estimate all the necessary parameters of the structural equation model (SEM), including correlations and variances to assess the study objectives. Assuming a Subjects-To-Variables (STV) ratio of 20:1 it will be possible to include up to 13 factors of influence (with 3 parameters per factor) into the model. Assuming a small variability in measured parameters in healthy volunteers, with respect to the parameters of interest, a number of 100 healthy volunteers is considered sufficient. |
| **Statistical methods** | Structural equation modelling (SEM) offers the possibility to analyse data with collinearities between the variables. Statistically, it represents an extension of General Linear Modelling (GLM) procedures, such as the ANOVA and multiple regression analysis. It is applicable to both experimental and non-experimental data, as well as to cross-sectional and longitudinal data. SEM takes a confirmatory (hypothesis testing) approach to the multivariate analysis of a s tructural theory, one that stipulates causal relations among multiple variables. Among the strengths of SEM is the ability to construct latent variables: variables which are not measured directly, but are estimated in the model from several measured variables each of which is predicted to “tap into” the latent variables. This allows the modeller to explicitly capture the unreliability of measurement in the model, which in theory allows the structural relations between latent variables to be accurately estimated. Factor analysis, path analysis and regression all represent special cases of SEM. It is important to note that SEM is more general than regression. In particular, a v ariable can act as both independent and dependent variable. Two main components of models are distinguished in SEM: the structural model showing potential causal dependencies between endogenous and exogenous variables, and the measurement model showing the relations between latent variables and their indicators. Exploratory and confirmatory factor analysis models, for example, contain only the measurement part, while path diagrams can be viewed as a SEM that only has the structural part. |

**CONTENTS**

1. [BACKGROUND INFORMATION AND STUDY RATIONALE 16](#_bookmark0)
2. [STUDY OBJECTIVES 20](#_bookmark1)
3. [STUDY DESIGN 21](#_bookmark2)
4. [SUBJECT SELECTION CRITERIA 21](#_bookmark3)
   1. [Subject recruitment 21](#_bookmark4)
   2. [Inclusion criteria 21](#_bookmark5)
   3. [Exclusion criteria 22](#_bookmark6)
   4. [Subject Withdrawals 23](#_bookmark7)
5. [STUDY PLAN 23](#_bookmark8)
   1. [Study Schedule 23](#_bookmark9)
   2. [Investigations 30](#_bookmark11)
6. [HEALTHCARE RESOURCE CONSUMPTION ASSESSMENTS 36](#_bookmark14)
7. [COLLECTION, RECORDING AND REPORTING OF SAFETY DATA 36](#_bookmark15)
8. [DATA MANAGEMENT 37](#_bookmark16)
9. [STATISTICAL METHODS 37](#_bookmark17)
   1. [Sample size 37](#_bookmark18)
   2. [Statistical analysis 38](#_bookmark19)
10. [ETHICS COMMITTEE/INSTITUTIONAL REVIEW BOARD APPROVAL 39](#_bookmark20)
11. [REGULATORY REQUIREMENTS 39](#_bookmark21)
12. [INFORMED CONSENT 39](#_bookmark22)
13. [DIRECT ACCESS TO SOURCE DOCUMENTS/DATA 39](#_bookmark23)
14. [STUDY MONITORING 39](#_bookmark24)
15. [QUALITY ASSURANCE 40](#_bookmark25)
16. [INSURANCE AND INDEMNITY 40](#_bookmark26)
17. [CONFIDENTIALITY 40](#_bookmark27)
18. [PREMATURE TERMINATION OF THE STUDY 40](#_bookmark28)
19. [CLINICAL STUDY REPORT 40](#_bookmark29)
20. [RECORD RETENTION 41](#_bookmark30)
21. [PUBLICATION OF RESULTS 41](#_bookmark31)
22. [REFERENCES 42](#_bookmark32)

[APPENDIX I 48](#_bookmark116)

## BACKGROUND INFORMATION AND STUDY RATIONALE

Asthma is a major public health problem with an estimated 300 million affected individuals worldwide [[1](#_bookmark33)]. Asthma is a chronic inflammatory disorder of the airways where inflammation and pathobiology derangements are present throughout the respiratory system [[2](#_bookmark34)[,3](#_bookmark35)[,4](#_bookmark36)]. The peripheral area of the lung with airways of < 2 mm diameter, termed small airways, constitutes a challenging compartment of the respiratory system as it is difficult to assess either physiologically or structurally in living subjects. Small airways can be affected by inflammation, remodelling like smooth muscle hyperplasia, and changes of the surrounding tissue, all contributing to small-airways dysfunction. Although recent studies suggest that small airway abnormalities contribute to the clinical expression of asthma [[5](#_bookmark37)], their role to asthma control and exacerbations has been minimally investigated.

Currently, several tests are available to assess different aspects of small-airways dysfunction and the value and limitations of each test have been recently extensively reviewed [[6,](#_bookmark38)[7,](#_bookmark39)[8](#_bookmark40)]. The conclusion of these reviews is that there is no gold standard tool to assess small airway dysfunction, and therefore, the available measurements provide indicative rather than conclusive information on the peripheral district. The most appropriate test or combination of tests to assess small airways still needs to be identified. It is also not known whether small airways dysfunction is present and the extent of involvement across all severity stages of asthma.

Small airways function has been measured in several ways using physiologic and radiographic testing in addition to direct and indirect assessments of small airways inflammation.

The **pulmonary function** tests that are used to assess small airway pathology can be subdivided in tests measuring flow, airway resistance, inhomogeneity of ventilation distribution, hyperinflation or air trapping. Flow measures commonly used in small airway studies are forced expiratory flow rates at 50% of vital capacity (FEF_50%_) and at 25-75% (FEF_25-75%_) of vital capacity [[9](#_bookmark41)]. Airway resistance can be measured with impulse oscillometry (IOS) and small airway obstruction is associated with an increase in resistance predominantly at lower frequencies [[10,](#_bookmark42)[11](#_bookmark43)]. Using IOS, Takeda et al. have shown that small airways dysfunction, as reflected by a higher R5- R20, was associated with symptoms of wheezing, dyspnoea and chest tightness [[12](#_bookmark44)]. When the severity of hyperresponsiveness was measured using both forced expiratory volume in the first second (FEV_1_) and IOS (R5-R20 and X5) during a methacholine challenge, a total of 9 out of 33 asthma patients developed symptoms without showing a fall in FEV_1_ [[13](#_bookmark45)]. In contrast to FEV_1_, R5- R20 and X5 increased at the time of symptom development, suggesting that an increase in small airways resistance is coupled with symptom development. This observation was consistent with the observation that increased small airways reactance is associated with more severe respiratory symptoms [[14](#_bookmark46)]. Furthermore, Downie and colleagues showed that small airway obstruction as assessed by multiple breath nitrogen washout (MBNW) contributed to the severity of hyperresponsiveness [[15](#_bookmark47)].

Lung volume assessment can inform on the presence of small airway dysfunction. Common measures of hyperinflation are functional residual capacity (FRC), residual volume (RV), total lung capacity (TLC) and the RV/TLC ratio measured with body plethysmography [[16](#_bookmark48)]. In addition, a larger difference between slow inspiratory vital capacity (SVC) and forced vital capacity (FVC) and the FVC to SVC ratio may be surrogate markers of the collapsibility of small airways [[17](#_bookmark49)]. Certainly, increased FRC or thoracic gas volume (TGV), TLC and RV greater than 120% predicted were associated with small airway obstruction in a study of lung volumes in over 4,000 p atients [[18](#_bookmark50)]. In a cohort of mild asthmatic subjects, the reduction in air trapping as determined by a significant decrease in RV, correlated with improvement in wheezing and shortness of breath [[19](#_bookmark51)].

To directly determine if hyperinflation is associated with small airways disease in asthma, transbronchial biopsy was performed in a cohort of mild to moderate asthmatic subjects who were not taking ant-inflammatory treatment controller therapy, which showed that TGV positively correlated with distal lung inflammation [[20](#_bookmark52)]. Thus, lung volumes offer an opportunity to evaluate distal lung parameters related to small airway inflammation and symptoms.

Inhomogeneity of peripheral ventilation distribution can be assessed by the multiple breath nitrogen washout test (MBNW) after inhalation of 100% O_2_. This test has the advantage over IOS that it does not only assess whether there is increased resistance in the small airways, but it is also able to dissect this into the more peripheral (S_acin_) and more central small a irway compartment (S_cond_). Using this MBNW test Farah *et al* have shown that a reduction in ventilation heterogeneity after treatment with inhaled corticosteroids (ICS) was the most important independent predictor of improvement in asthma control [[21](#_bookmark53)]. This data are in line with other findings showing that that the severity of hyperresponsiveness is associated with the severity of small airway obstruction as measured by multiple breath nitrogen washout test [[22](#_bookmark54)].

Clearly, all these lung function measurements can inform on small airways dysfunction. However, no study has been conducted longitudinally in the same subjects, and the data regarding the most reliable lung function test or combination of tests reflecting small airways disease in asthma are currently not conclusive. Moreover, these tests have only been used in small numbers of asthmatic subjects and not over the full spectrum of asthma severity, thus hampering our ability to determine if small airway obstruction is equally present over all severities of asthma.

Direct **evaluation of inflammation** via lung biopsy remains the gold standard to assess the inflammatory profile and to compare it with physiologic tests of small airways function. Unfortunately, transbronchial biopsy (TBBX), the most effective method to assess small airway inflammation in living patients with asthma, is an invasive procedure that carries a low risk of pneumothorax of approximately 1% [[23](#_bookmark55)]. Due to this risk, only a few studies have been performed using this technique. The specimens obtained by TBBX contain both alveolar and small airway tissue, and the amount of each to quantify inflammation is limited as the total volume of recovered tissue is small. Despite these barriers, several studies have documented increased inflammation in the distal lung compartment, which includes the small airways and alveolar tissue as generally the quantification of inflammatory cells combined both compartments [[2](#_bookmark34)[,3](#_bookmark35)[,24](#_bookmark56)]. In a study of asthmatic subjects undergoing thoracic surgery, larger lung samples were analysed and revealed significant inflammation throughout the lung, either in large and small airways as assessed by eosinophil and mast cells markers [[25](#_bookmark57)].

Indirect measurements of inflammation that are used in clinical practice include fractional exhaled nitric oxide (FeNO), induced sputum and serum biomarkers. Measurement of FeNO is a quantitative, non-invasive, simple, and safe method of measuring airway inflammation in asthma [[1](#_bookmark33)]. The technique is easy to perform and can be repeated even in patients with severe airflow obstruction. Patients with asthma have high levels of NO in their exhaled breath and high levels of inducible nitric oxide synthase enzyme expression in airway epithelial cells. Importantly, FeNO decreases in response to treatment with corticosteroids [[26](#_bookmark58)]. Two compartment models of pulmonary NO production have been described [[27](#_bookmark59)], which can be used to calculate the bronchial (J_NO_) and alveolar contribution (C_alv_) to exhaled NO concentration [[28](#_bookmark60)]. Calv is elevated in conditions associated with distal lung inflammation, such as alveolitis [[29](#_bookmark61)] and chronic obstructive pulmonary disease [[30](#_bookmark62)] and has been related to bronchoalveolar lavage eosinophil cationic protein levels in asthmatic children [[31](#_bookmark63)] and to bronchoalveolar lavage eosinophil counts in asthmatic adults [[32](#_bookmark64)]. A study in asthmatic patients showed that an extrafine formulation of an ICS able to reach the small airways, lowers both bronchial and alveolar exhaled NO, whereas the conventional (non-extrafine particle) formulation of the same ICS affected only bronchial NO [[33](#_bookmark65)]. This suggests that in asthmatic subjects C_alv_ may inform on the inflammatory process of the peripheral district.

Increasing attention is being given to the role of **biomarkers** to improve diagnosis and management of asthma, particularly of the most severe expressions of the disease where the therapeutic need is more compelling [[34,](#_bookmark66)[35](#_bookmark67)].

Serum total immunoglobulin E (IgE) level has been the first biomarker routinely used to assess indications and doses for the monoclonal IgE-specific antibody omalizumab in patients inadequately controlled despite daily high doses of ICS and LABA. Sputum eosinophil count has been tested as biomarker of the response to anti-inflammatory treatments: two studies have found that the frequency of exacerbations in severe patients can be reduced when ICS dose is adjusted to keep sputum eosinophil count within the ‘‘normal’’ range [[36](#_bookmark68)[,37](#_bookmark69)]. In recent clinical trials, antibodies blocking IL-5 (with its crucial role in the growth, maturation and activation of eosinophils) decreased the frequency of asthma exacerbations in patients with severe, exacerbation- prone, eosinophilic asthma as assessed by sputum and/or blood eosinophilia (or surrogates) [[38](#_bookmark70)[,39](#_bookmark71)[,40](#_bookmark72)]. Other biologics are currently under different stages of investigation, including molecules directed against IL-4, IL-13, IL-9, GM-CSF and TNFα, and the related biomarkers are thoroughly searched [[41](#_bookmark73)]. Recently, the IL-13-specific monoclonal antibody lebrikizumab has been found to be more effective in moderate/severe patients with high serum levels of periostin, an IL-13-inducible extracellular matrix protein [[42](#_bookmark74)]. This implies that easily detectable biomarkers such as periostin could be routinely used in clinical practice to identify specific asthmatic patients potentially responsive to therapeutic strategies targeting a specific pathway. Whether these, and/or additional biomarkers currently under assessment, have specific relationships or target pathogenetic mechanisms related to small airways dysfunction as assessed by the different tools (lung function, imaging, inflammation, clinical questionnaires) adopted in this study has never been previously explored.

With regard to imaging, advances in computed tomography (CT) technology and software analysis platforms have amplified **imaging** resolution to detailed quantitative analyses of airway geometry [[43,](#_bookmark75)[44](#_bookmark76)]. CT scanning cannot be used for the direct evaluation of the dimensions of the small airways as they are beyond the currently available resolution. However, indirect changes caused by the small airways on the lung parenchyma can be detected by CT, as small airways dysfunction results in reduced ventilation of part of the lung, which in turn induces a reflex reduction in perfusion that is highlighted as areas of decreased attenuation on thoracic CT images [[44](#_bookmark76)[,45](#_bookmark77)]. Heterogeneity of lung attenuation in asthma can be noticeably accentuated in expiratory scans compared to inspiratory CT scans, due to regional differences in small airway closure or emptying rate. Although asymptomatic individuals with no l ung function abnormalities also demonstrate low attenuation regions on CT scans, [[46](#_bookmark78)] asthmatic patients have significantly more air-trapping which correlates with lung function abnormalities [[47,](#_bookmark79)[48,](#_bookmark80)[49,](#_bookmark81)[50](#_bookmark82)]. Air trapping has been shown to correlate with asthma severity [[51](#_bookmark83)], yet the relationship is not fully understood. CT assessed air-trapping has also been associated with airway hyper-responsiveness [[50](#_bookmark82)], disease duration [[48](#_bookmark80)], airflow limitation [[48](#_bookmark80)[,49](#_bookmark81)[,50](#_bookmark82)] and used for evaluation of response to inhaled corticosteroid therapy [[52](#_bookmark84)]. Moreover, Zeidler et al [[53](#_bookmark85)] showed that asthmatic patients allergic to cat had increased air trapping at expiratory CT scan 6 hours after cat exposure, while this was not measurable with FEV1 values, suggesting that small airways obstruction was significantly present despite normal spirometric values. The state-of-the-art imaging approach to assess the small airways in a multicentre study is therefore inspiratory and expiratory CT-derived densitometry indices carefully standardized for extra-thoracic air, and blood and regression equations calculated from the scanning of densitometry standards embedded within lung phantoms. This strategy provides detailed measures of air-trapping coupled to proximal airway geometry and allows for later analyses using novel approaches such as ‘Parametric Response Mapping’ [[54](#_bookmark86)].

Asthma is a complex disease wherein multiple genes and their interaction with environmental factors contribute to disease development [[55](#_bookmark87)]. Genomic DNA can be obtained from

peripheral blood and assessed for **genetic studies**. There are two main approaches to identify genes predisposing/linked to diseases. The candidate gene approach tests genes that have been selected for their relevance to the pathophysiology of the disease. With this approach a group of genes has been replicated many times and consistently associated with asthma in different meta-analyses, including *ADAM33, IL13, IL4RA, TNF,* and *TBXA2R*. These genes may represent common major asthma genes [[56](#_bookmark88)]. At variance to candidate gene approaches, asthma genome-wide association studies (GWAS) are hypothesis-free, because the markers that are set throughout the genome are merely used to identify loci associated with the disease and are not selected based on t heir assumed biologic functions. The most recent multi-center GWAS provide strong statistical evidence for the association of many genes, including the *IKZF3-ZPBP2-GSDMB-ORMDL3* locus, *HLA-DQ*, *IL1RL1*, *IL18RL1*, *IL33*, *TSLP*, *SLC22A5*, *SMAD3*, and *RORA*, with asthma [[57](#_bookmark89)[,58](#_bookmark90)]. Whether these genes and related pathways can predict or correlate with small airways dysfunction as assessed by the different perspectives/tools (lung function, imaging, inflammation, clinical questionnaires) adopted in this study to investigate this field has never been previously explored.

It has been shown that gene expression profiling in bronchoscopically brushed bronchial epithelial cell can be a useful tool to investigate the underlying pathophysiology of asthma [[59](#_bookmark91)]. In addition, it can be used a biomarker to predict corticosteroid responsiveness in patients with asthma [[60](#_bookmark92)]. Importantly, it has been shown that gene expression changes in bronchial epithelium, e.g. after smoking, are closely correlated to gene expression changes in nasal epithelium [[61](#_bookmark93)]. A major advantage of **nasal brushings** over bronchial brushings is that they can be obtained in a non- invasive way.

Lastly, use of Patient Reported Outcomes (PROs) such as questionnaires has gained prominence as a method to better quantify asthma symptoms longitudinally [[62](#_bookmark94)]. Well-validated self-assessment questionnaires have been developed to monitor the level of asthma control, such as the asthma control questionnaire (ACQ-6) and asthma control test (ACT) [[35,](#_bookmark67)[63](#_bookmark95)]. Among questionnaires to evaluate disease-specific quality of life, the Asthma Quality of Life Questionnaire (AQLQ) was developed to measure the functional problems (physical, emotional, social and occupational) that are most troublesome to adults with asthma [[64](#_bookmark96)]. Additionally, EuroQol-5D is a standardised measure of health status developed by the EuroQol Group in order to provide a simple, generic measure of health for clinical and economic appraisal [[65](#_bookmark97)]. Applicable to a wide range of health conditions and treatments [[66](#_bookmark98)], it provides a simple descriptive profile and a single index value for health status that can be used in the clinical and economic evaluation of health care as well as in population health surveys. EuroQol-5D-5L will be used in the present study to compare healthy subject with asthma subjects as this measure can assess quality of life in both in relation to the other measures of small airway function specifically to delineate normal from abnormal. Recently, Van der Molen et al have developed a questionnaire aiming to quantify symptoms of bronchial hyperresponsiveness in patients with asthma, the bronchial hyperresponsiveness questionnaire (BHQ). This test has been used in a double blind study and appeared to relate to changes in objectively tested hyperresponsiveness [[67](#_bookmark99)]. This is of interest to indirectly assess hyperresponsiveness and associate this with small airway dysfunction in the current study since small airways dysfunction was independently of large airway obstruction associated with the severity of bronchial hyperresponsiveness [[68](#_bookmark100)]. Whether these questionnaires can predict or correlate with small airways function is not known as there is no que stionnaire that specifically addresses small airways function that can be used by the general practitioners or pulmonary specialists. To address this issue, Van der Molen et al recently developed a s mall airway dysfunction tool (SADT) to assess the presence of small airway dysfunction on a patient level in asthma (data not published yet). SADT will be validated in the current study both cross-sectionally and longitudinally for its use in assessing whether patients have small airway dysfunction.

Lastly, as long as asthma severity and clinical outcomes are affected by adherence to pharmacological treatment, a medication adherence scale will be administered during the study. The Morisky Medication Adherence Scale (MMAS-8) is a validated questionnaire that estimates the risk of medication non-adherence which was previously used [[69](#_bookmark101)].

Therefore, the overall hypothesis of this study is that small airways disease (SAD) in asthma significantly contributes to asthma pathobiology, and measures of small airways function will correlate with asthma control and exacerbations. To test this hypothesis, we will recruit 800 subjects with mild, moderate and severe asthma, and 100 healthy controls (to provide normal reference values for the study variables). We will follow them longitudinally with periodic assessments to determine small and large airway function by using physiologic and radiographic techniques as well as direct and indirect measures of inflammation above described. We will determine if either of these tests or combination of tests not only define small airways dysfunction in asthma, but whether SAD modulates asthma control and risk of exacerbations.

The trial will be carried out in conformity with the Code of Federal Regulations (21 CFR 50) and the Declaration of Helsinki (as applicable, with attention being drawn to Section concerning freely given consent), Good Clinical Practices and with all the other local laws and regulations relevant to non-pharmacological intervention studies.

## STUDY OBJECTIVES

The **ATLANTIS** (**A**ssessmen**T** of smal**L A**irways involveme**NT I**n a**S**thma) trial aims are:

*Major objectives:*

- To determine the role of small airways abnormalities in the clinical manifestations of asthma;
- To evaluate which (combination of) clinical methods best assesses the abnormalities of small airways and large airways disease in asthma and best relates to asthma severity, control, and future risk of exacerbations, both cross-sectionally and longitudinally;
- To assess if a questionnaire (Small Airways Dysfunction Tool) could be offered to physicians in diagnosing Small Airways Disease (SAD) in asthma, and thus characterize asthma patients with small airways diseases as determined by physiologic and radiographic assessments and measurement of specific biomarkers.

*Minor objectives:*

- To define the physiologic characteristics that correlate with small airways function in asthma as compared to healthy controls.
- To define the radiographic characteristics that correlate with small airways function in asthma as compared to healthy controls.
- To determine which direct and indirect measures of inflammation best discriminate between the large and small airway compartments.
- To determine if questionnaires such as ACQ-6 and ACT assess small airways function.
- To determine the correlation between SAD and asthma control.
- To determine if SAD is associated with exacerbations requiring prescription of oral corticosteroids.

## STUDY DESIGN

This is a multinational, multicentre, non-pharmacological intervention study made up of a cross-sectional and a longitudinal phase. The Investigator is directly or indirectly (via GP) responsible for the appropriate individual treatment for the subject. The assignment of the patient to a particular therapeutic strategy falls within current practice and the prescription of the medicine is clearly separated from the decision to include the patient in the study. The study is not intended to collect information on a single, specific drug. The prescribed treatment will be reported with the trade or generic name of each component.

The study plan foresees 3 clinic visits and 2 t elephone contacts during a 1-year observation period. Asthmatic patients and healthy controls satisfying all the inclusion and none of the exclusion criteria will enter the study cross-sectional phase visit (**V1**, baseline visit) and the 12- month longitudinal phase. Follow-up visits to apply the study procedures (see Table 1: Study Schedule) will take place after 6 (**V2**) and 12 (**V3**) months from the cross-sectional phase visit (**V1**, baseline visit). In addition, after 3 and 9 months from baseline, all subjects will be contacted by phone calls for the evaluations described in Table 1. The end of the trial is defined as the last visit of the last subject in the trial.

## SUBJECT SELECTION CRITERIA

### Subject recruitment

900 subjects divided into 800 asthmatic patients and 100 healthy controls, will be required. Recruitment will be balanced, at the extent possible, in order to achieve at least > 150 patients each for steps 1 - 4 and > 50 patients for step 5 as indicated in the international guidelines (GINA 2012) on the basis of their current therapy.

The 100 healthy controls will be recruited on the basis of absence of respiratory symptoms, airway obstruction and hyperresponsiveness.

### Inclusion criteria

- - 1. **Asthmatic patient inclusion criteria**

1. Male or female patients aged ≥ 18 and ≤ 65, who have signed an Informed Consent form prior to initiation of any study-related procedure.
2. Clinical diagnosis of asthma for at least 6 months confirmed by a chest physician according to international guidelines (GINA 2012) supported by objective evidence of any of the following at the baseline visit or in the previous 5 years:
   1. Positive response to methacholine challenge test [PC_20_ < 8 mg/mL or PD_20_ < 0.7 mg for those subjects not using inhaled corticosteroids (ICS) and PC_20_ < 16 mg/mL or PD_20_ < 1.4 mg for subjects using ICS]

#### or

- 1. Positive response to a reversibility test, defined as ΔFEV_1_ ≥ 12% and ≥ 200 mL over baseline FEV_1_, within 30 minutes after administration of 400 μg of salbutamol pMDI administered with or without Spacer

#### or

- 1. Peak Flow variability (i.e. highest - lowest PEF over the day/mean value of the two,

× 100) > 20%, measured over a follow-up period of 7 days

#### or

- 1. Documented response (defined as ΔFEV_1_ ≥ 12% and ≥ 200 mL) after a cycle (e.g., 4 weeks) of regular maintenance anti-asthma treatment.

1. Patients with stable asthma, on any previous regular asthma treatment (“rescue” β_2_-agonists alone included) at a stable dose, for at least 8 weeks prior to baseline visit.
2. Current smoker, ex-smoker (since the past 12 months) or lifelong non-smoker (total lifetime smoking history < 10 pack-years defined as [(number of cigarettes smoked per day)x(number of years of smoking)] / 20).
   - 1. **Healthy subject inclusion criteria**
3. Male or female patients aged ≥ 18 and ≤ 65, who have signed an Informed Consent form prior to initiation of any study-related procedure.
4. No clinical history of asthma or COPD (no respiratory symptoms compatible to asthma or COPD in the past 2 years).
5. Current smoker, ex-smoker (since the past 12 months) or lifelong non-smoker (total lifetime smoking history < 10 pack-years).
6. Normal spirometry: baseline FEV_1_ ≥ 80% of the predicted normal value, FEV_1_/FVC > LLN (lower limit of normal).
7. Normal airways responsiveness: PC_20_ ≥ 16 mg/mL, PD_20_ ≥ 1.4 mg.

### Exclusion criteria

- - 1. **Asthmatic patient exclusion criteria**

The presence of any of the following will exclude a subject from study enrolment:

1. Cigarette smoking > 10 pack-years defined as [(number of cigarettes smoked per day)x(number of years of smoking)] / 20).
2. Clinical diagnosis of COPD confirmed by a chest physician.
3. Asthma exacerbation in the 8 weeks prior to baseline visit (see [section 5.2.1](#_bookmark11)).
4. Clinical or functional uncontrolled respiratory, haematological, immunologic, renal, neurologic, hepatic, endocrinal or other disease, or any condition that might, in the judgment of the investigator, compromise the results or interpretation of the study.
5. Pregnant or lactating women (a urine pregnancy test will be performed).
6. Participation in an interventional clinical trial with intake of the last dose of any investigational drug <12 weeks preceding baseline visit (last dose < 5 half-lives prior to baseline visit for biologics).
7. Inability to comply with study procedures.
8. Alcohol or drug abuse.
   - 1. **Healthy subject exclusion criteria**

The presence of any of the following will exclude a subject from study enrolment:

1. Cigarette smoking history > 10 pack-years defined as [(number of cigarettes smoked per day)x(number of years of smoking)] / 20.
2. Diagnosed upper and/or lower respiratory tract diseases.
3. Clinical or functional uncontrolled haematological, immunologic, renal, neurologic, hepatic, endocrinal or other disease, or any condition that might, in the judgment of the investigator, compromise the results or interpretation of the study.
4. Pregnant or lactating women (a urine pregnancy test will be performed).
5. Participation in an interventional clinical trial with intake of the last dose of any investigational drug <12 weeks preceding baseline visit (last dose < 5 half-lives prior to baseline visit for biologics).
6. Inability to comply with study procedures.
7. Alcohol or drug abuse.

### Subject Withdrawals

Subjects have the right to withdraw from the study at any time for any reason, including personal reasons.

The investigator also has the right to withdraw subjects from the study in the event of:

- A medical occurrence which is considered intolerable by the patient or the Investigator;
- Non-adherence with the protocol and/or lack of willingness or commitment to co-operate in all phases of the study;
- Development of an exclusion criterion (apart from smoking and asthma exacerbations);
- Inclusion in another clinical trial with an investigational drug during the course of the present study;
- Although there are no risks related to an investigational drug, because the assignment of the patient to a particular therapeutic strategy falls within current practice and the prescription of the medicine is clearly separated from the decision to include the patient in the study, in case of pregnancy during the study, the subject can be withdrawn from the study procedures at discretion of the Investigator in order to define the best schedule of visits in the subject’s interest;
- Abnormal test results that the investigator considers clinically significant and warranting the withdrawal of the patient from the study (e.g. *it is the responsibility of the local site to review the CT scans clinically and if there are abnormalities that require further investigations then these are undertaken locally as per local guidelines and according to clinical practice*);
- The sponsor or the regulatory authorities or the Ethics Committee(s), for any reason, terminates the entire study, or terminates the study for this trial site or this particular subject.

It is understood by all concerned that an excessive rate of withdrawals can render the study uninterpretable, therefore unnecessary withdrawals of subjects should be avoided. However, should a subject decide to withdraw for other reasons, all efforts will be made to complete and report the observations as thoroughly as possible. The investigator must fill-in the “Study Termination Form” in the e-CRF explaining all reasons for withdrawal. Whenever a p atient withdraws his or her consent, all of his or her samples collected will be destroyed, if requested.

## STUDY PLAN

### Study Schedule

The study plan foresees:

- A baseline visit [visit 1 (**V1**)], divided into 2 or 3 days, during which asthmatic patients and healthy controls will be selected and will enter the 12-month study period.
- Two follow-up visits [visit 2 ( **V2**) and visit 3 ( **V3**)] after 6 and 12 months from baseline visit, respectively.
- Two follow-up phone contacts after 3 (phone contact 1) and 9 (phone contact 2) months from baseline visit.

Visit 1 w ill be done on 2 separate days (1a and 1b) with a “window” of a maximum of 5 days between visits 1a and 1b. O nly in case of subjects included in the subgroup undergoing bronchoscopy with endobronchial and transbronchial biopsy, a third day (1c) will be scheduled with a “window” of maximum of 4 weeks from visit 1b. For visits 2 and 3 and the Follow up Phone Contacts after 3 and 9 months from V1 a “window” of -15 to +15 days is allowed (calculated from the last day of visit 1).

The study plan and scheduled tests are summarized in the following flow-chart:

| **Table 1: Study Schedule** | Visit 1  *Study entry* | | | Phone contact 1  Month 3  (±15 days) | Visit 2  Month 6  (±15 days) | Phone contact 2  Month 9  (±15 days) | Visit 3  Month 12  (±15 days) |
| --- | --- | --- | --- | --- | --- | --- | --- |
|  | **1a** | **1b** | **1c** |  |  |  |  |
| Written informed consent |  |  |  |  |  |  |  |
| Inclusion criteria |  |  |  |  |  |  |  |
| Exclusion/withdrawal criteria |  |  |  | β(1) | β(1) | β(1) | β(1) |
| Medical history/ongoing medications |  |  |  |  |  |  |  |
| Antiasthma medications^(2)^ |  |  |  |  |  |  |  |
| Asthma exacerbations^(2)^ |  |  |  |  |  |  |  |
| Physical examination |  |  |  |  |  |  |  |
| Urine pregnancy test^(3)^ |  |  |  |  |  |  |  |
| Collection, recording and reporting of Safety data |  |  |  |  |  |  |  |
| Blood sample collection^(4)^ |  |  |  |  |  |  |  |
| Phadiatop™ test |  |  |  |  |  |  |  |
| Healthcare resource consumption assessments ^(2)^ |  |  |  |  |  |  |  |
| Morisky Medication Adherence Scale (MMAS-8) ^(2)^ |  |  |  |  |  |  |  |
| Asthma Control Test (ACT)^(2)^ |  |  |  |  |  |  |  |
| Small Airways dysfunction Tool (SADT) |  |  |  |  |  |  |  |
| Impulse Oscillometry (IOS)^(5)^ |  | β**(6)** |  |  |  |  |  |
| Fraction of exhaled NO (FeNO)^(5)^ |  |  |  |  |  |  |  |

| Multiple-Breath Nitrogen Washout (MBNW) |  | β**(6)** |  |  |  |  |  |
| --- | --- | --- | --- | --- | --- | --- | --- |
| Body plethysmographic measurements |  | β**(6)** |  |  |  |  |  |
| Spirometric measurements |  | β**(6)** |  |  |  |  |  |
| Sputum induction^(7)^ |  |  |  |  |  |  |  |
| Nasal brushing^(7)^ |  |  |  |  |  |  |  |
| Methacholine challenge test^(8)^ |  |  |  |  |  |  |  |
| Asthma Control Questionnaire (ACQ-6)^(2)^ |  |  |  |  |  |  |  |
| Asthma Quality of Life Questionnaire (mini- AQLQ)^(2)^ |  |  |  |  |  |  |  |
| Standardised measure of health status descriptive system (EuroQol-5D-5L) |  |  |  |  |  |  |  |
| Bronchial Hyper responsiveness questionnaire (BHQ)^(9)^ |  |  |  |  |  |  |  |
| Computed tomography (CT) scan^(10)^ |  |  |  |  |  |  |  |
| Bronchoscopy with endobronchial and trans-bronchial biopsy^(11)^ |  |  |  |  |  |  |  |

*^(1)^ Apart from smoking and asthma exacerbations*

*^(2)^ Only in asthmatics.*

*^(3)^ Dipstick test performed in all women physiologically capable of becoming pregnant UNLESS post-menopausal [12 months of natural (spontaneous) amenorrhea], or have evidence of surgical sterilization (e.g., bilateral tubal ligation, hysterectomy).*

*^(4)^ Blood differential and total cell counts: evaluation by Local Laboratory. At least 1 hour fasting should be respected. For those subjects who consented for the optional collection of serum, plasma and blood cells, sample to be shipped and stored at Central Laboratory for future analysis on the pathobiology of asthma.*

*^(5)^ Measurements to be done only if the instrument is locally available at the site.*

*^(6)^ Both pre- and post-bronchodilator measurements (the latter only in asthmatics)*

*^(7)^ Only in a subset of subjects in selected sites in EU, US and Canada; collection of samples and/or evaluation by Central Laboratories.*

*^(8)^ Methacholine challenge test will be performed only in subjects with a baseline FEV_1_ greater than 55% predicted [70].*

*^(9)^ UK, US and The Netherlands only.*

*^(10)^ CT scans will be done in selected sites only in a subgroup of ~ 530 asthmatic patients balanced, at the extent possible, into the five treatment groups (see section 4.1 “Subject recruitment”) as indicated in the international guidelines (GINA) and 50 healthy controls (580 subjects in total). Central processing will be conducted. It is the responsibility of the local site to review the scans clinically and i f there are abnormalities that require further investigations then these are undertaken locally as per local guidelines and according to clinical practice.*

*^(11)^ Only in specialized sites.*

- - 1. **Visit 1 (baseline visit)**

A baseline visit (Visit 1) will be carried out in order to enrol eligible consenting subjects in the study.

Visit 1 will be done on 2 separate days (1a and 1b) with a “window” of max 5 days in between. Only in case of subjects included in the subgroup undergoing bronchoscopy with endobronchial and trans-bronchial biopsy, a third day (1c) will be scheduled with a “window” of max 4 weeks from visit 1b.

#### Visit 1a

The following procedures will be performed in the first day of Visit 1 (1a) in the following order:

- - - - - written informed consent from the patient, after the study has been fully explained by the Investigator or delegated study staff personnel;
        - Review of inclusion/exclusion criteria;
        - The patient will be identified by a number of seven digits. The first two digits will identify the country, the second two digits the site number, and the last three digits the patient’s number (patient’s ID) sequentially assigned to each patient of each site by a chronological attribution (i.e. for the first patient selected they will be 001, 002 for the second patient and so on);
        - Recording of medical history (only ongoing and chronic diseases should be reported as the study is prospective) and ongoing medications for concomitant diseases;
        - History of asthma exacerbations (see [section 5.2.1](#_bookmark11)) in the last year (asthmatics only);
        - Asthma medications (asthmatics only);
        - Adherence to medications for asthma will be evaluated with MMAS-8 (asthmatics only);
        - Full physical examination including vital signs;
        - Urine pregnancy test (dipstick): performed in all women physiologically capable of becoming pregnant UNLESS post-menopausal [12 months of natural (spontaneous) amenorrhea], or have evidence of surgical sterilization (e.g., bilateral tubal ligation, hysterectomy);
        - Blood sample collection after at least 1 hour fasting (patient must be informed) in order to perform routine haematology and phadiatop test (see [section 5.2](#_bookmark12).4);
        - Blood sample collection for future analysis on the pathobiology of asthma (optional, applicable only in EU, US and Canada sites);
        - Healthcare resource consumption assessments (asthmatics only);
        - ACT, ACQ-6 and mini-AQLQ will be administered to asthmatic patients;
        - SADT and EuroQol-5D-5L will be administered to both asthmatic patients and healthy controls;
        - BHQ will be administered to both asthmatic patients and healthy controls in selected sites only (United Kingdom, US and The Netherlands);
        - FeNO where applicable, depending on the availability of the equipment at the site;
        - Methacholine challenge test will be performed in subjects with a b aseline FEV1 >55% predicted;
        - Nasal brushing– selected sites in EU, US and Canada;
        - Collection, recording and reporting of Safety data (see [section 7](#_bookmark15)).

An appointment for the second day of visit 1 (1b) will be taken within 5 days from the first day of visit 1 (1a) in the morning.

#### Visit 1b

The following procedures will be performed in the second day of Visit 1 ( 1b) in the following order:

- - - - - Review of inclusion/exclusion criteria;
        - Pre-bronchodilator IOS where applicable, depending on the availability of the equipment at the site;
        - Pre-bronchodilator Multiple Breath Nitrogen Washout Test (MBNWT);
        - Pre-bronchodilator lung volumes measurement with body box;
        - Pre-bronchodilator spirometry;
        - Administration of 4 puffs (4 × 100 µ g) of salbutamol pMDI plus spacer (the following testing starts 15 minutes after salbutamol inhalation, only in asthmatics);
        - Post-bronchodilator IOS where applicable, depending on the availability of the equipment at the site (only in asthmatics);
        - Post-bronchodilator Multiple Breath Nitrogen Washout Test (MBNWT);
        - Post-bronchodilator lung volumes measurement with body box;
        - Post-bronchodilator spirometry;
        - Sputum induction – selected sites in EU, US and Canada;
        - A CT scan will be performed in a subgroup of subjects only in pre-defined selected sites;
        - An appointment for the phone contact will be taken in 3 months’ (±15 days) time. Asthmatic patients will be provided with a blank copy of ACT, ACQ-6, mini-AQLQ, MMAS-8 and EuroQol-5D-5L, whereas healthy controls will be provided with blank copy of EuroQol-5D-5L only;
        - Collection, recording and reporting of Safety data (see [section 7](#_bookmark15)).

An appointment for the next clinic visit will be taken in 6 months (±15 days).

An appointment for the third day of visit 1 (1c) will be taken within 4 weeks from the second day of visit 1 (1b) in the morning only in case of subjects included in the subgroup undergoing bronchoscopy with endobronchial and trans-bronchial biopsy.

#### Visit 1c

The following procedures will be performed in the third day of Visit 1 (1c) only in case of subjects included in the subgroup undergoing bronchoscopy with endobronchial and trans-bronchial biopsy:

- - - - - Review of inclusion/exclusion criteria;
        - Urine pregnancy test (dipstick): performed in all women physiologically capable of becoming pregnant UNLESS post-menopausal [12 months of natural (spontaneous)

amenorrhea], or have evidence of surgical sterilization (e.g., bilateral tubal ligation, hysterectomy);

- - - - - Bronchoscopy with endobronchial and transbronchial biopsy will be performed in a subgroup of subjects only in pre-defined selected sites;
        - Collection, recording and reporting of Safety data (see [s](#_bookmark13)[ection 7](#_bookmark15)).
    1. **Phone contact 1 [month 3 (±15 days)]**

The following procedures will be performed during the phone contact:

- Asthma medications taken by the patient will be reviewed and changes from last visit will be recorded in the CRF (asthmatics only);
- Occurrence of asthma exacerbations (see [section 5.2.1](#_bookmark11)) since the last visit will be reviewed (asthmatics only);
- Review of withdrawal criteria;
- Asthma patients will be asked to fill in the ACT, ACQ-6, mini-AQLQ, EuroQol-5D-5L questionnaires and bring back the originals at the next visits at the site;
- Healthy controls will be asked to fill in the EuroQol-5D-5L only and bring back the originals at the next visits at the site;
- Adherence to medications for asthma will be evaluated with MMAS-8 (asthmatics only);
- Healthcare resource consumption assessments (asthmatics only).
- Collection, recording and reporting of Safety data (see [section 7](#_bookmark15)).

Subjects will be asked to bring back the original paper questionnaires at the next clinic visit at the site (previously scheduled).

- - 1. **Visit 2 [month 6 (±15 days)]**

The following procedures will be performed in Visit 2 in the following order:

- Collection of paper copy of the questionnaires filled-in during the previous phone contact;
- Review of withdrawal criteria;
- Full physical examination including vital signs;
- Blood sample collection after at least 1 hour fasting (patient must be informed) in order to perform routine haematology;
- Blood sample collection for future analysis on the pathobiology of asthma (optional, applicable only in EU, US and Canada sites);
- Asthma medications (asthmatics only);
- Adherence to medications for asthma will be evaluated with MMAS-8 (asthmatics only);
- Healthcare resource consumption assessments (asthmatics only);
- Occurrence of asthma exacerbations (see [section 5.2.1](#_bookmark11)) since the last visit will be reviewed (asthmatics only);
- ACT, ACQ-6 and mini-AQLQ, will be administered to asthmatic patients;
- SADT and EuroQol-5D-5L will be administered to both asthmatic patients and healthy controls;
- BHQ will be administered to both asthmatic patients and healthy controls in selected sites only (United Kingdom, US and The Netherlands);
- FeNO and IOS measurements where applicable, depending on t he availability of the equipment at the site;
- Multiple Breath Nitrogen Washout Test (MBNWT);
- Lung volumes measurement with body box;
- Spirometry;
- Nasal brushing – selected sites in EU, US and Canada;
- Collection, recording and reporting of Safety data (see [section 7](#_bookmark15)).

An appointment for the phone contact will be taken in 3 months’ (±15 days) time. Asthma patients will be provided with a blank copy of ACT, ACQ-6, mini-AQLQ, MMAS-8 and EuroQol-5D-5L, whereas healthy controls will be provided with blank copy of EuroQol-5D-5L only.

An appointment for the next clinic visit will be taken in 6 months’ (±15 days)

- - 1. **Phone contact 2 [month 9 (±15 days)]**

The following procedures will be performed during the phone contact:

- Asthma medications taken by the patient will be reviewed and changes from last visit will be recorded in the CRF (asthmatics only);
- Occurrence of asthma exacerbations (see [section 5.2.1](#_bookmark11)) since the last visit will be reviewed (asthmatics only);
- Revision of withdrawal criteria;
- Asthma patients will be asked to fill in the ACT, ACQ-6, mini-AQLQ, EuroQol-5D-5L questionnaires and bring back the originals at the next visits at the site;
- Healthy controls will be asked to fill in the EuroQol-5D-5L only and bring back the originals at the next visits at the site;
- Adherence to medications for asthma will be evaluated with MMAS-8 (asthmatics only);
- Healthcare resource consumption assessments (asthmatics only);
- Collection, recording and reporting of Safety data (see [section 7](#_bookmark15)).

Subjects will be asked to bring back the original paper questionnaires at the next clinic visit at the site (previously scheduled).

- - 1. **Visit 3 [month 12 (±15 days)]**

The following procedures will be performed in Visit 3 in the following order:

- Collection of paper copy of the questionnaires filled-in during the previous phone contact;
- Revision of withdrawal criteria;
- Full physical examination including vital signs;
- Blood sample collection after at least 1 hour fasting (patient must be informed) in order to perform routine haematology;
- Blood sample collection for future analysis on the pathobiology of asthma (optional, applicable only in EU, US and Canada sites);
- Asthma medications taken by the patient will be reviewed and changes from last visit will be recorded in the CRF (asthmatics only);
- Adherence to medications for asthma will be evaluated with MMAS-8 (asthmatics only);
- Healthcare resource consumption assessments (asthmatics only);
- Occurrence of asthma exacerbations (see [section 5.2.1](#_bookmark11)) since the last visit will be reviewed (asthmatics only);
- ACT, ACQ-6 and mini-AQLQ will be administered to asthmatic patients;
- SADT and EuroQol-5D-5L will be administered to both asthmatic patients and healthy controls;
- BHQ will be administered to both asthmatic patients and healthy controls in selected sites only (United Kingdom, US and The Netherlands);
- FeNO and IOS measurements where applicable, depending on t he availability of the equipment at the site;
- Multiple Breath Nitrogen Washout Test (MBNWT);
- Lung volumes measurement with body box;
- Spirometry;
- Collection, recording and reporting of Safety data (see [section 7](#_bookmark15)).

### Investigations

All measurements will be performed according to international guidelines, if available (e.g. spirometry), or pre-specified, standardized procedures to be found in the study-specific Working Procedures (WP) Manual. All measurements are well-known standardized and approved procedures, none is investigational apart from the validation of the questionnaire Small Airways Dysfunction Tool (SADT).

- - 1. **Asthma exacerbations**

An "asthma exacerbation" will be defined as a significant deterioration of asthma and signalled by any or more of the following:

- *need for a systemic corticosteroid course (≥ 3 days);*
- *hospitalisation for asthma;*
- *emergency room attendance for asthma.*

Medical records will be requested from patients to document their exacerbations if the evaluation took place in a healthcare provider’s office. Information will be collected on whether treatment for exacerbation was prescribed by a general practitioner, a m edical specialist or self-administered. Subjects will be followed longitudinally to determine if the alterations in small airways function are related to poor asthma control and increased risk of exacerbations.

In the present study, exacerbations will be collected retrospectively at each clinic visits and during phone contacts. Patients should be informed to bring medical documentation about their exacerbations at the extent possible and/or report to the study staff information about exacerbations.

- - 1. **Measurements of lung physiology**

#### Spirometry and body plethysmography

Lung function measurements and daily calibration of the equipment will be done according to the recommendation of the Official Statement of the European Respiratory Society and American Thoracic Society [[71](#_bookmark102)]. Predicted values will be calculated according to the formulas reported by Quanjer et al. [[72](#_bookmark103)]. Throughout the study, the clinic visits and the lung function measurements will start in the morning, approximately at the same time of the day for each patient. Lung function measurements will be performed after appropriate washout from bronchodilators.

The following parameters will be assessed at clinic visits (1b, 2 and 3), full details are reported in the WP Manual:

- Forced Expiratory Volume in the 1st second (FEV1, L);
- Forced Vital Capacity (FVC, L);
- Forced Expiratory Flow between 25% and 75% of Vital Capacity (FEF25%, FEF50%, FEF75%, FEF25%-75%, L/sec).
- Slow Inspiratory Vital Capacity (IVC, L);
- Residual Volume (RV, L);
- Total Lung Capacity (TLC, L);
- Functional Residual Capacity (FRC, L);
- “Effective” Airway Resistance (Raw, kPa * s / L);
- “Effective” Specific Airway Conductance (sGaw, 1/ kPa * s).

In the present study, spirometry and body plethysmography will be performed during each clinic visit. Post-bronchodilator measurements will be performed only in visit 1b, whereas measurement of lung function in visits 2 and 3 will be only pre-bronchodilator.

#### Impulse Oscillometry System (IOS)

The impulse oscillometry system (IOS) has been proposed as a simple, sensitive, and noninvasive method to assess pulmonary function without forced manoeuvres [[73](#_bookmark104)] and has been increasingly used to assess resistance and reactance in asthmatic patients. IOS is a technique used to measure respiratory resistance (R) and reactance (X) at different frequencies and can provide separate measurements for both large and small airways function [[10](#_bookmark42)].

The following parameters will be assessed (full details are reported in the WP Manual):

- Resistance (R5, R20, R5-20, kPa L s^-1^);

-1

-1

- Reactance (X5, kPa L

s^-1^).

-1 -1

- Area of reactance (AX, Hz kPa L s ).

In the present study, IOS will be performed during each clinic visit (1b, 2 and 3), only if the instrument is locally available at the site.

#### Multiple-Breath Nitrogen Washout Test (MBNWT)

Marked ventilation distribution abnormalities occur in obstructive lung disease despite normal ventilator capacity as measured by spirometry. Washout tests, such as the MBNWT, may provide insight into mechanisms behind abnormal ventilation distribution and localisation of pathology [[74](#_bookmark105)]. MBNWT assesses ventilation distribution inhomogeneity during tidal breathing

from FRC, by examining inert gas clearance over a s eries of breaths and requires only passive cooperation and minimal coordination. The indices of ventilation heterogeneity in the peripheral regions of the lung, where gas transport occurs predominantly across a pressure gradient through convection [ventilation heterogeneity in convection-dependent airways (*S_cond_*)] and across a concentration gradient through diffusion [ventilation heterogeneity in diffusion-dependent airways (*S_acin_*)], are derived [[22](#_bookmark47)]. The upper limit of normal for *S_cond_* is 0.037 L^-1^, and that for *S_acin_* is 0.130 L^-1^. The reproducibility for the same parameters are ±0.026 L^-1^ for *S_cond_* and ±0.027 L^-1^ for *S_acin_* [[75](#_bookmark106)].

In the present study, MBNWT will be performed during each clinic visit (1b, 2 a nd 3). Full details for the parameters to assess are reported in the WP Manual.

#### Methacholine challenge test

The methacholine challenge test [[76](#_bookmark107)] will be performed at visit 1a according to standard procedures described in full in the study-specific WP Manual.

- - 1. **Questionnaires**

#### Asthma Control Test (ACT)

Asthma control is important to assess in clinical practice, although it is multidimensional in nature, characterized by symptoms, changes in pulmonary function, and effects on quality of life and functional ability [[63,](#_bookmark95)[77](#_bookmark108)]. The ACT survey is a patient-completed questionnaire with 5 items assessing asthma symptoms (daytime and nocturnal), use of rescue medications, and the effect of asthma on daily functioning. Each item includes 5 response options corresponding to a 5-point Likert-type rating scale. In scoring the ACT survey, responses for each of the 5 items are summed to yield a score ranging from 5 ( poor control of asthma) to 25 (complete control of asthma).

In the present study, the ACT will be administered at visits 1a, 2 and 3.

Moreover, the Investigator will remind patients to complete the questionnaire at home during phone contacts.

#### 6-item Asthma Control Questionnaire (ACQ-6)

The 6-item ACQ includes a m easure of the top 5 asthma symptoms (woken at night by symptoms, day-time symptoms, limitation of daily activities, shortness of breath and wheeze) and the use of quick-relief bronchodilators. Subjects will be asked to recall how their asthma has been during the previous week and to respond to the symptom and use questions on a 7-point scale (0 = no impairment, 6 = maximum impairment). The questions are equally weighted and the ACQ score is the mean of the scores to the 6 que stions and therefore between 0 (totally controlled) and 6 (severely uncontrolled). The 6-item ACQ is applicable to all adults with asthma and is reliable and reproducible when the clinical state is stable and able to discriminate between patients with different levels of asthma control [[35](#_bookmark67)[,78](#_bookmark109)]. The asthma control level will be evaluated according to the following cut-off:

- Controlled asthma: ACQ score < 0.75;
- Partly controlled asthma: ACQ score 0.75 - 1.5;
- Uncontrolled asthma: ACQ score ≥ 1.5.

In the present study, the ACQ-6 will be administered at visits 1a, 2 and 3.

Moreover, the Investigator will remind patients to complete the questionnaire at home during phone contacts.

#### Mini Asthma Quality of Life Questionnaire (mini-AQLQ)

The mini-AQLQ contains 15 questions in 4 domains: symptoms, activity limitation, emotional function and environmental stimuli. Patients are asked to think about how they have been during the previous two weeks and to respond to each of the 15 que stions on a 7-point scale (7=no impairment; 1=severe impairment). The overall score is the mean of the scores to all responses and the individual domain scores are the means of the scores to the items in those domains. It takes 3-4 minutes to complete [[79](#_bookmark110)].

In the present study, the mini-AQLQ will be administered at visits 1a, 2 and 3.

Moreover, the Investigator will remind patients to complete the questionnaire at home during phone contacts.

#### Standardised measure of health status descriptive system (EuroQol-5D-5L)

EuroQol-5D is a standardised measure of health status developed by the EuroQol Group in order to provide a simple, generic measure of health for clinical and economic appraisal [[65,](#_bookmark97)[66](#_bookmark98)]. Applicable to a wide range of health conditions and treatments, it provides a simple descriptive profile and a single index value for health status that can be used in the clinical and economic evaluation of health care as well as in population health surveys. EuroQol-5D is designed for self-completion by respondents and is ideally suited for use in postal surveys, in clinics, and in face-to-face interviews. It is cognitively undemanding, taking only a few minutes to complete. Instructions to respondents are included in the questionnaire.

The new version of the EuroQol-5D includes five levels of severity in each of the existing five EuroQol-5D dimensions and it is called the EuroQol-5D-5L [[65,](#_bookmark97)[66](#_bookmark98)], consisting of 2 pages – the EuroQol-5D-5L descriptive system and the EQ visual Analogue scale (EQ VAS). The descriptive system comprises 5 dimensions (mobility, self-care, usual activities, pain/discomfort, anxiety/depression), each dimension having 5 l evels: no pr oblems, slight problems, moderate problems, severe problems, and extreme problems. The respondent is asked to indicate his/her health state by ticking (or placing a cross) in the box against the most appropriate statement in each of the 5 dimensions. This decision results in a 1-digit number expressing the level selected for that dimension. The digits for 5 dimensions can be combined in a 5-digit number describing the respondent’s health state. It should be noted that the numerals 1-5 have no arithmetic properties and should not be used as a cardinal score. The EQ VAS records the respondent’s self- rated health on a 20 cm vertical, visual analogue scale with endpoints labelled ‘the best health you can imagine’ and ‘the worst health you can imagine’. This information can be used as a quantitative measure of health as judged by the individual respondents. The EuroQol-5D-5L asks respondents to simply ‘mark an X on the scale to indicate how your health is TODAY’ and then to ‘write the number you marked on the scale in the box below’. This should make the task easier for both respondents and users.

In the present study, the EuroQol-5D-5L will be administered at visits 1a, 2 and 3.

Moreover, the Investigator will remind patients to complete the questionnaire at home during phone contacts.

#### Small Airways Dysfunction Tool (SADT)

A preliminary 63- item small airways dysfunction tool (SADT) aiming to identify patients with SAD has been developed that includes items based on differences between asthma patients with and without SAD [[80](#_bookmark111)]. Items cover a broad area of asthma signs and symptoms related to severity of allergies, hyperresponsiveness and relationship with physical exercise. In the present study, the SADT will be administered to all subjects at visits 1a, 2 and 3.

#### Bronchial Hyperresponsiveness Questionnaire (BHQ)

BHQ assesses the presence and severity of bronchial hyper responsiveness (BHR) in asthma according to FDA-approved standards for the development of questionnaires. This BHR Questionnaire (BHQ) is a condition-specific questionnaire, consisting of 34 items, 15 regarding symptoms and 19 r egarding provoking stimuli [[67](#_bookmark99)]. This is the first questionnaire specifically developed for the assessment of BHR in asthma. By using the PC_20_-histamine as the main reference, the expert panel [[67](#_bookmark99)] selected items for the BHQ thereby expecting the BHQ to be a condition-specific instrument. Various attempts have been made in the past to find a relationship between symptoms and/or stimuli that provoke respiratory symptoms on the one hand, and the severity or presence of BHR on the other. Studies investigating this subject used questionnaires such as the International Union against Tuberculosis and Lung Disease (IUATLD) questionnaire, the Medical Research Council (MRC) questionnaire, the American Thoracic Society-Division of Lung Diseases (ATSDLD) questionnaire, or a subset of questions from these questionnaires. Although some questions derived from these questionnaires show a relation with BHR, so far no specific instrument has been developed to measure patient-perceived BHR. Items related to BHR were generated by asthma patients. The expert panel decided to choose mild criteria for the item reduction. In this way they wished to prevent loss of possible predictive items. A disadvantage of this method is the relatively large number (n=34) of questions selected for the BHQ. In the present study, the BHQ will be administered to all subjects at visits 1a, 2 and 3 (selected sites in United Kingdom, US and The Netherlands).

#### 8-item Morisky Medication Adherence Scale (MMAS-8)

The MMAS is a generic assessment of medication-taking behaviour and has been used in a number of health conditions, both chronic and infectious diseases. It consists of 8 items assessing reasons for non-adherence [[69](#_bookmark101)]. The theory underlying this measure was that failure to adhere to a medication regimen could occur because of several factors such as “do you sometimes have problems remembering to take your medication?” “do you sometimes forget to take your medication?” and problems with the complexity of the medical regimen such as, “do you ever feel hassled about sticking to your treatment plan?” The questions are phrased to avoid the “yes- saying” bias by reversing the wording of the questions about the way patients might experience failure in following their medication regimen, since there is a tendency for patients to give their physicians or other health care providers positive answers. Each item measures a specific medication-taking behavior and not a determinant of adherence behaviour. Response categories are yes/no for each item with a dichotomous response and a 5-point Likert response for the last item.

In the present study, the MMAS will be administered at visits 1a, 2 and 3.

Moreover, the Investigator will remind patients to complete the questionnaire at home during phone contacts.

- - 1. **Measurement of Inflammation and Biomarkers**

#### Blood drawn

Blood will be drawn at each clinic visit (1a, 2 and 3) for local total and differential cell count. Phadiatop testing will be perfomed in visit 1a.

Optional serum, plasma and blood cells collection will be applicable only in EU, US and Canada sites. For those subjects who consented for the optional collection of serum, plasma and blood cells, samples will be sent to the Central Laboratory (INTERLAB central lab services GmbH) and stored for future analysis (including genetic tests on D NA) on t he pathobiology of asthma

(asthma-related biomarkers) which will be defined in future specific protocols. Standard procedures for blood processing are described in full in the study-specific WP Manual.

#### Sputum induction – selected sites only (EU, US and Canada)

Sputum induction will be performed in visit 1b. Processing will be done according to standard procedures as described in full in the study-specific WP Manual.

Sputum cytospin slides will be sent to Pulmonary Research Institute (Grosshansdorf, Germany) for differential cell count analysis. Optional collection of sputum supernatant will be also applicable. For those subjects who consented for the optional collection, sputum supernatant will be sent to the Central Laboratory (INTERLAB central lab services GmbH) and stored for future analysis on the pathobiology of asthma. The analyses will be defined in future specific protocols.

#### Nasal brushing – selected sites only (EU, US and Canada), optional assessment

For those subjects consenting for this optional assessment, RNA will be extracted in visits 1a and 2 and will be sent to the Central Laboratory (INTERLAB central lab services GmbH). Cells will be cryopreserved for future analysis on the pathobiology of asthma if the sample is adequate. The analyses will be defined in future specific protocols. Standard procedures for nasal brushing sampling are described in full in study-specific WP Manual.

#### Fractional Exhaled Nitric Oxide (FeNO)

In the present study, FeNO will be performed during each clinic visit (1b, 2 and 3), only if the instrument is locally available at the site. Depending on the specific equipment available at the site FeNO will be measured as standard single flow (50ml/s) or multiple flows. Standard procedures for FeNO measurements are described in full in the study-specific WP Manual.

#### Bronchoscopy with endobronchial and transbronchial biopsy (optional)

Visit 1c: to be performed in specialized centres (selected sites).

Those subjects who consented will undergo the optional bronchoscopy with endobronchial (EBBX) and transbronchial (TBBX) biopsies as previously described [[3](#_bookmark35)] and also described in the Bronchoscopy Manual of Procedures.

The location of the biopsies will be the right lower lobe. Prior to the bronchoscopies the subjects will undergo spirometry before and after inhalation of albuterol from a metered dose inhaler (MDI). Lidocaine will be used to anesthetize the upper airway and will also be applied to the laryngeal area, trachea, and orifice of the right lower or left lower lobe bronchi via the bronchoscope.

Five TBBX will be performed under fluoroscopic guidance followed by five EBBX performed under direct visualization from the fourth and fifth generation airways. Supplemental oxygen will be administered throughout the procedure along with monitoring of heart rate and oxygen saturation. A chest radiograph will be performed after each procedure to rule out the presence of a pneumothorax. Subjects will be monitored in the laboratory for at least 2 h after the procedure. Endobronchial (EBBX) and transbronchial biopsy (TBBX) will be either fixed, embedded and sectioned, or processed for RNA extraction. The tissue will be stained for analysis of the total number of inflammatory cells and quantification of specific cells including eosinophils, neutrophils, T cells and macrophages. Part of the fixed tissue will be sectioned but not stained for later evaluation which could include quantification of other inflammatory cells, assessment of airway matrix to determine presence of a particular mediator.

Gene expression profiles (including RNA) will be performed at a later date for future analysis on the pathobiology of asthma which will be defined in future specific protocols.

Staining and imaging on fixed tissue will be done at the University of California, Davis Computational Core, USA. Quantification of imaging data and long term storage of all biopsy

tissues and samples (both fixed and processed for RNA extraction) will be done at Duke University, Durham, USA.

Standard procedures for bronchoscopy and biopsy are described in full in the study-specific Bronchoscopy Manual of Procedures.

- - 1. **Imaging**

Visit 1b, c entral processing: thoracic computed tomography (CT) scan in a subgroup of ~ 530 asthmatic patients balanced, at the extent possible, into the five treatment groups (see section 4.1 “Subject recruitment”) as indicated in the international guidelines (GINA) and 50 healthy controls (580 subjects in total). Internationally there are a l ack of normal ranges for CT-derived airway geometry and densitometry. For this reason approximately 50 h ealthy controls will undergo CT scan to provide reference normal values to help interpret differences observed in disease.

Full detail about how to acquire the CT scans and how data will be analysed is reported in the study-specific WP Manual.

Effective radiation dose will be calculated at each site by each scanner, according to the local National regulations.

It is the responsibility of the local site to review the scans clinically and if there are abnormalities that require further investigations then these are undertaken locally as per local guidelines and according to clinical practice. CT scans will be performed in a subgroup of patients in selected sites only.

## HEALTHCARE RESOURCE CONSUMPTION ASSESSMENTS

- Asthma-specific of hospital admissions (number and length);
- Asthma-specific emergency room or urgent care visits;
- Unscheduled consultations for asthma (without hospitalisation) defined as: the need for a visit (Medical Specialist or General Practitioner) due to symptoms worsening.
- Unscheduled tests for asthma (without hospitalisation).

## COLLECTION, RECORDING AND REPORTING OF SAFETY DATA

It is responsibility of the Investigator to collect all Adverse Drug Reactions (ADRs) derived by spontaneous, unsolicited reports of subjects, by observation and by routine open questionings (e.g., how have you felt since I saw you last? Is there anything new that you wish to discuss? Have you experienced any side effects with the treatment you use?).

An ADR is defined as “a response to a medicinal product which is noxious and unintended” (Directive 2010/84/EU amending Article 1(11) of Directive 2001/83/EC). “Response in this context means that a c ausal relationship between a m edicinal product and an adverse event is at least a reasonable possibility” (ICH-E2A Guideline). ADRs may arise from use of the product within or outside the terms of the marketing authorisation or from occupational exposure. Conditions of use outside the marketing authorisation include off-label, overdose, misuse, abuse and medication errors (“Special cases”). All ADRs occurring during the study must be reported to the Authorities according to the applicable laws. The Investigator (or reporting physician) is also recommended to report all ADRs to the relevant Marketing Authorisation Holders of the involved medicinal products. Additionally, it should be reported also conditions of use outside the marketing authorisation of the medicinal products (i.e. off-label, overdose, misuse, abuse and medication errors) or from occupational exposure, as well as cases of suspected drug interaction, pregnancy, breast-feeding exposure and lack of efficacy.

For ADRs related to Chiesi Farmaceutici S.p.A.’s medicinal products, the following contact details should be used.

**The contact details of the Sponsor’s Safety Contact:**

**Full name**: Castiglione Gian Nicola, MD

**Title**: Director, Corporate Pharmacovigilance, EU Qualified Person Responsible for Pharmacovigilance

**Address**: Via Palermo 26/A 43122 Parma – Italy

**Telephone**: +39 0521 279 701

**Fax**: +39 0521 271 992

**e-mail**: [cds@chiesi.com](mailto:cds@chiesi.com)

## DATA MANAGEMENT

An electronic CRF (e-CRF) will be filled-in by the Investigator and/or his/her designee. Front-end edit checks will run at the time of data collection and back-end edit checks will be used by the Data Manager to check for discrepancies and to ensure consistency and completeness of the data. Medical history will be coded using the MedDRA dictionary; medications will be coded using the WHO Drug dictionary. External data will be processed centrally and results will be sent electronically to the designated CRO.

After cleaning of data, once the database has been declared to be complete and accurate, it will be locked and the planned statistical analysis will be performed. After database lock, well-documented updates to the study data are possible only if an error is found in the data, that is critical and has an impact on the overall results/outcome of the analysis. A CD-ROM of the subject data will be sent after database lock at the investigational site for archiving.

## STATISTICAL METHODS

The statistical analysis will be performed by CROS NT s.r.l. (Verona, Italy). This section provides specifications for the preparation of the final SAP, which will be issued prior to database lock. Any differences compared to this statistical section should be identified and documented in the final SAP. Data summarization and statistical analyses will be performed using the SAS System, Version

9.2 for PC or later.

### Sample size

800 patients will be sufficient to estimate all the necessary parameters of the structural equation model (SEM), including correlations and variances to assess the study objectives. Assuming a Subjects-To-Variables (STV) ratio of 20:1 [[81,](#_bookmark112) [82](#_bookmark113)] it will be possible to include up to13 factors of influence (with 3 parameter per factor) into the model. Assuming a small variability in measured parameters in healthy volunteer, with respect to the parameters of interest, a number of 100 healthy volunteers is considered sufficient.

The sample size for the number of patients undergoing CT-scans is calculated based on the following assumptions:

- incidence of patients with SAD is expected to be approx. 20%;
- agreement between classifications as SAD due to the SEM and CT-scan should be at least

0.8 (Cohen’s kappa [[83](#_bookmark114)]);

- error probability, Type-I-error, should be less than 0.05 and
- Power should be at least 80%.

Following the algorithm given by A. Cantor [[84](#_bookmark115)] sample size of not less than 528 patients valid for CT-scan and SEM evaluation is calculated.

Moreover, approximately 50 healthy subjects will undergo CT scan to provide reference normal values for airways geometry and densitometry.

### Statistical analysis

Structural equation modelling (SEM) offers the possibility to analyse data with collinearities between the variables. Statistically, it represents an extension of General Linear Modelling (GLM) procedures, such as the ANOVA and multiple regression analysis. It is applicable to both experimental and non-experimental data, as well as to cross-sectional and longitudinal data. SEM takes a confirmatory (hypothesis testing) approach to the multivariate analysis of a structural theory, one that stipulates causal relations among multiple variables. Among the strengths of SEM is the ability to construct latent variables: variables which are not measured directly, but are estimated in the model from several measured variables each of which is predicted to “tap into” the latent variables. This allows the modeller to explicitly capture the unreliability of measurement in the model, which in theory allows the structural relations between latent variables to be accurately estimated. Factor analysis, path analysis and regression all represent special cases of SEM. It is important to note that SEM is more general than regression. In particular, a variable can act as both independent and dependent variable. Two main components of models are distinguished in SEM: the structural model showing potential causal dependencies between endogenous and exogenous variables, and the measurement model showing the relations between latent variables and their indicators. Exploratory and confirmatory factor analysis models, for example, contain only the measurement part, while path diagrams can be viewed as a SEM that only has the structural part.

The classifications for patients as having SAD or not as well from SEM as from CT-scans will be compared in order to evaluate the rate of agreement, using Cohen’s kappa coefficient [[83](#_bookmark114), [84](#_bookmark115)].

The variables included in the analyses will be specified in the Statistical Analysis Plan (SAP).

- - 1. **Descriptive Statistics**

Descriptive statistics will be provided in summary tables by treatment group according to the type of variable summarised:

- for quantitative variables: standard quantitative statistics (N, mean, standard deviation, median, minimum and maximum)
- for qualitative variables: frequency distribution [number of non-missing observations (N) and percentages (%)].

#### Subjects accountability

Disposition of subjects, subject status and subjects excluded from analysis sets will be summarized. Also data from withdrawn subjects will be considered for appropriate statistical analysis.

- - 1. **Missing data**

Further details on dealing with missing data, along with the handling of possible outliers, will be described in the SAP.

- - 1. **Patient demographics and baseline characteristics**

Descriptive statistics will be presented at baseline (cross sectional phase) in order to describe the population characteristics at study entry.

## ETHICS COMMITTEE/INSTITUTIONAL REVIEW BOARD APPROVAL

The study proposal will be submitted to the Ethics Committee/Institutional Review Board in accordance with the requirements of each country.

The EC/IRB shall give its opinion in writing -clearly identifying the study number, study title and informed consent form approved-, before the clinical trial commences.

A copy of all communications with the EC/IRB will be provided to the Sponsor.

The Investigator should provide written reports to the EC/IRB annually or more frequently if requested on any changes significantly affecting the conduct of the trial and/or increasing risk to the subjects (according to the requirements of each country).

## REGULATORY REQUIREMENTS

The study will be notified to the Health Authorities (or authorized by) according to the legal requirements in each participating country*.*

Selection of the subjects will not start before the approval of the Ethics Committee/Institutional Review Board has been obtained and the study notified to Health Authorities (or authorized by) where required.

The study will be conducted in accordance with the Declaration of Helsinki, the Code of Federal Regulations (21 CFR 50) ,the Good Clinical Practices guidelines and following all other requirements of local laws.

## INFORMED CONSENT

It is the responsibility of the Investigator to obtain written consent from each subject or from the subject’s legal representative prior to any study related procedures taking place.

If the subject and his/her legal representative are unable to read, the informed consent will be obtained in the presence of an impartial witness, e.g., a person independent of the study who will read the informed consent form and the written information for the subject.

Consent must be documented by the subject’s dated signature. The signature confirms that the consent is based on information that has been understood. Moreover, the Investigator must sign and date the informed consent form.

Each subject’s signed informed consent must be kept on file by the Investigator. One copy must be given to the subject.

## DIRECT ACCESS TO SOURCE DOCUMENTS/DATA

The Investigators or designated must permit trial-related monitoring, audits, Ethics Committee/Institutional Review Board review or regulatory inspection, providing direct access to source data/documents.

## STUDY MONITORING

Monitoring will be performed by CROMSOURCE who has been designated by Chiesi.

It is understood that the monitor(s) will contact and visit the Investigator/centre before the study, regularly throughout the study and after the study had been completed, and that they will be permitted to inspect the various study records: case reports form, Investigator study file and source data (source data is any data that is recorded elsewhere to the case report forms), provided that subject confidentiality is respected.

The purposes of these visits are:

- to assess the progress of the study;
- to review the compliance with the study protocol and WP manual;
- to discuss any emergent problem;
- to validate the contents of the e-CRFs against the source documents (see [Appendix I](#_bookmark116) for the minimum list of source data required);
- prior to each monitoring visit, the Investigator or staff will record all data generated since the last visit on the case report forms. The Investigator and/or study staff will be expected to be available for at least a portion of the monitoring visit to answer questions and to provide any missing information.
- it is possible that the Investigator site may be audited by Sponsor personnel or regulatory national and/or international regulatory agencies during and after the study has been completed.

## QUALITY ASSURANCE

The R&D Quality Assurance Department of Chiesi may perform an audit at any time according to the Sponsor’s Standard Operating Procedures, in order to verify whether the study is being conducted in agreement with Good Clinical Practices.

## INSURANCE AND INDEMNITY

Chiesi holds and will maintain an adequate insurance policy covering damages arising out of Chiesi’s sponsored clinical research studies.

Chiesi will indemnify the Investigator and hold him/her harmless for claims for damages arising out of the investigation (including study procedures, e.g. biopsies or CT scans), in excess of those covered by his/her own professional liability insurance, providing that the investigations were performed under his/her or deputy’s supervision and in strict accordance with accepted medical practice and with the study protocol.

The Investigator must notify Chiesi immediately upon notice of any claims or lawsuits.

## CONFIDENTIALITY

All study documents are provided by the Sponsor in confidence to the Investigator and his/her appointed staff. None of this material may be disclosed to any party not directly involved in the study without written permission from Chiesi.

The Investigator must assure the subject’s anonymity will be maintained. The Investigator will keep a separate list with at least the initials, the subject’s study numbers, names, and (optional) addresses and telephone numbers. The Investigator will maintain this for the longest period of time allowed by his/her own institution and, in any case, until further communication from Chiesi.

## PREMATURE TERMINATION OF THE STUDY

Both the Sponsor and the Investigator reserve the right to terminate the study at any time. Should this be necessary, the procedures for an early termination or temporary halt will be arranged after consultation by all involved parties.

The Sponsor should submit a written notification to the Regulatory Authority concerned and Ethics Committee/Institutional Review Board providing the justification of premature ending or of the temporary halt.

## CLINICAL STUDY REPORT

The clinical study report, including the statistical and clinical evaluations, shall be prepared and sent to co-ordinating Investigator’s for agreement and signature.

At the end of the trial a summary of the clinical study report will be provided to all Ethics Committees/Institutional Review Boards, to the Competent Authority where applicable and to Investigators.

## RECORD RETENTION

After completion of the study, all the documentation relating to the identification and subsequent therapy of the subjects enrolled in the study, as well as all the original documentation of the study (signed study protocol and signed protocol amendments, notification to the EC etc.), together with copies of the CRFs, will be stored in the Clinical Centre archives, until Sponsor request and in agreement with national and international regulations.

It is the responsibility of the Sponsor to inform the Investigator of when these documents can be destroyed. The Investigator must contact Chiesi before destroying any trial-related documentation. In addition, all subjects’ medical records and other source documentation will be kept for the maximum time permitted by the institution.

## PUBLICATION OF RESULTS

Chiesi is entitled to publish and/or present any results of this study at scientific meetings, and to submit the clinical trial data to national and international Regulatory Authorities. Chiesi furthermore reserves the right to use such data for industrial purposes.

Investigators will inform Chiesi and the Study Steering Committee before using the results of the study for publication or presentation, and agree to provide the Sponsor with a copy of the proposed presentation. Data from individual study sites must not be published separately.

Negative as well as positive results should be published or otherwise made publicly available.

## REFERENCES

1. Global Initiative for Asthma (GINA): Global Strategy for Asthma Management and Prevention: NHLBI/WHO workshop report. Bethesda: National Institutes of Health, National Heart, Lung and Blood Institute; 2002. Publication No. 02-3659: 1-116. Last Update 2009.
2. Kraft M, Djukanovic R, Wilson S, Holgate ST, Martin RJ: Alveolar tissue inflammation in asthma. Am J Respir Crit Care Med 1996, 154:1505-1510.
3. Kraft M, Martin RJ, Wilson S, Djukanovic R, Holgate ST: Lymphocyte and eosinophil influx into alveolar tissue in nocturnal asthma. Am J Respir Crit Care Med 1999, 159(1):228-234.
4. Bergeron C, Hauber HP, Gotfried M Newman K, Dhanda R, Servi RJ, et al. J Allergy Clin Immunol. 2005;116:983-989.
5. van der Wiel E, ten Hacken NH, Postma DS, van den Berge M. Small-airways dysfunction associates with respiratory symptoms and clinical features of asthma: a systematic review. J Allergy Clin Immunol. 2013 Mar;131(3):646-57.
6. van den Berge M, ten Hacken NH, Cohen J, Douma WR, Postma DS. Small airway disease in asthma and COPD: clinical implications. Chest 2011;139: 412-23.
7. Contoli M, Bousquet J, Fabbri LM Magnussen H, Rabe KF, Siafakas NM, et al. The small airways and distal lung compartment in asthma and COPD: a time for reappraisal. Allergy 2010;65:141-51.
8. Scichilone N, Battaglia S, Olivieri D, Bellia V. The role of small airways in monitoring the response to asthma treatment: what is beyond FEV1? Allergy 2009;64: 1563-9.
9. Bar-Yishay E, Amirav I, Goldberg S. Comparison of maximal midexpiratory flow rate and forced expiratory flow at 50% of vital capacity in children. Chest. 2003 Mar;123(3):731-5.
10. Goldman MD. Clinical application of forced oscillation. Pulm Pharmacol Ther. 2001;14(5):341-50.
11. Hoshino M. Comparison of effectiveness in ciclesonide and fluticasone propionate on s mall airway function in mild asthma. Allergol Int. 2010 Mar;59(1):59-66.
12. Takeda T, Oga T, Niimi A, Matsumoto H, Ito I, Yamaguchi M, et al. Relationship between small airway function and health status, dyspnea and disease control in asthma. Respiration. 2010;80(2):120-6.
13. Segal LN, Goldring RM, Oppenheimer BW, Stabile A, Reibman J, Rom WN, et al. Disparity between proximal and distal airway reactivity during methacholine challenge. COPD. 2011 Jun;8(3):145-52.
14. Mansur AH, Manney S, Ayres JG. Methacholine-induced asthma symptoms correlate with impulse oscillometry but not spirometry. Respir Med. 2008 Jan;102(1):42-9.
15. Downie SR, Salome CM, Verbanck S, Thompson B, Berend N, King GG. Ventilation heterogeneity is a major determinant of airway hyperresponsiveness in asthma, independent of airway inflammation.Thorax. 2007 Aug;62(8):684-9.
16. van den Berge M, ten Hacken NH, Cohen J, Douma WR, Postma DS. Small airway disease in asthma and COPD: clinical implications. Chest. 2011 Feb;139(2):412-23.
17. Cohen J, Postma DS, Vink-Klooster K, van der Bij W, Verschuuren E, Ten Hacken NH, et al. FVC to slow inspiratory vital capacity ratio: a potential marker for small airways obstruction. Chest. 2007 Oct;132(4):1198-203.
18. Dykstra BJ, Scanlon PD, Kester MM, Beck KC, Enright PL. Lung volumes in 4,774 patients with obstructive lung disease. Chest. 1999 Jan;115(1):68-74.
19. Kraft M, Cairns CB, Ellison MC, Pak J, Irvin C, Wenzel S. Improvements in distal lung function correlate with asthma symptoms after treatment with oral montelukast. Chest. 2006 Dec;130(6):1726-32.
20. Sutherland ER, Martin RJ, Bowler RP, Zhang Y, Rex MD, Kraft M. Physiologic correlates of distal lung inflammation in asthma. J Allergy Clin Immunol. 2004 Jun;113(6):1046-50.
21. Farah CS, King GG, Brown NJ, Downie SR, Kermode JA, Hardaker KM, et al. The role of the small airways in the clinical expression of asthma in adults. J Allergy Clin Immunol. 2012 Feb;129(2):381-387.
22. Downie SR, Salome CM, Verbanck S, Thompson B, Berend N, King GG. Ventilation heterogeneity is a major determinant of airway hyperresponsiveness in asthma, independent of airway inflammation. Thorax. 2007 Aug;62(8):684-9.
23. Tukey MH, Wiener RS. Population-based estimates of transbronchial lung biopsy utilization and complications. Respir Med. 2012 Nov;106(11):1559-65.
24. Wenzel SE, Szefler SJ, Leung DY, Sloan SI, Rex MD, Martin RJ. Bronchoscopic evaluation of severe asthma. Persistent inflammation associated with high dose glucocorticoids. Am J Respir Crit Care Med. 1997 Sep;156(3 Pt 1):737-43.
25. Hamid Q, Song Y, Kotsimbos TC, Minshall E, Bai TR, Hegele RG, et al. Inflammation of small airways in asthma. J Allergy Clin Immunol. 1997 Jul;100(1):44-51.
26. Dweik RA, Boggs PB, Erzurum SC, Irvin CG, Leigh MW, Lundberg JO, et al. American Thoracic Society Committee on Interpretation of Exhaled Nitric Oxide Levels (FENO) for Clinical Applications. An official ATS clinical practice guideline: interpretation of exhaled nitric oxide levels (FENO) for clinical applications. Am J Respir Crit Care Med. 2011 S ep 1;184(5):602-15.
27. Tsoukias NM, and George SC. A two-compartment model of pulmonary nitric oxide exchange dynamics. J Appl Physiol 85:653– 666, 1998.
28. George SC, Hogman M, Permutt S, Silkoff. Modeling pulmonary nitric oxide exchange. J Appl Physiol 96:831– 839, 2004.
29. Lehtimaki L, Kankaanranta H, Saarelainen S, Hahtola P, Järvenpää R, Koivula T, et al. Extended exhaled NO measurement differentiates between alveolar and bronchial inflammation. Am J Respir Crit Care Med 163:1557– 1561, 2001.
30. Brindicci C, Ito K, Resta O, Pride NB, Barnes PJ, Kharitonov SA.. Exhaled nitric oxide from lung periphery is increased in COPD. Eur Respir J 26:52–59, 2005.
31. Mahut B, Delclaux C, Tillie-Leblond I, Gosset P, Delacourt C, Zerah-Lancner F, et al. Both inflammation and remodeling influence nitric oxide output in children with refractory asthma. J Allergy Clin Immunol 113:252–256, 2004.
32. Berry M, Hargadon B, Morgan A, Shelley M, Richter J, Shaw D, Green RH, et al. Alveolar nitric oxide in adults with asthma: Evidence of distal lung inflammation in refractory asthma. Eur Respir J 25:986 –991, 2005.
33. Nicolini G, Chetta A, Simonazzi A, Tzani P, Aiello M, Olivieri D. Both bronchial and alveolar exhaled nitric oxide are reduced with extrafine beclomethasone dipropionate in asthma. Allergy Asthma Proc. 2010 Sep-Oct;31(5):85-90.
34. Taylor DR, Pavord ID. Biomarkers in the assessment and management of airways diseases. Postgrad Med J. 2008 Dec;84(998):628-34.
35. Reddel HK, Taylor DR, Bateman ED, Boulet LP, Boushey HA, Busse WW, et al. American Thoracic Society/European Respiratory Society Task Force on Asthma Control and Exacerbations. An official American Thoracic Society/European Respiratory Society statement: asthma control and exacerbations: standardizing endpoints for clinical asthma trials and clinical practice. Am J Respir Crit Care Med. 2009 Jul 1;180(1):59-99.
36. Green RH, Brightling CE, McKenna S, Hargadon B, Parker D, Bradding P, et al. Asthma exacerbations and sputum eosinophil counts: a randomised controlled trial. Lancet. 2002 Nov 30;360(9347):1715-21.
37. Jayaram L, Pizzichini MM, Cook RJ, Boulet LP, Lemière C, Pizzichini E, Cartier A, Hussack P, Goldsmith CH, Laviolette M, Parameswaran K, Hargreave FE. Determining asthma treatment by monitoring sputum cell counts: effect on exacerbations. Eur Respir J. 2006 Mar;27(3):483-94.
38. Haldar P, Brightling CE, Hargadon B, Gupta S, Monteiro W, Sousa A, et al. Mepolizumab and exacerbations of refractory eosinophilic asthma. N Engl J Med. 2009 Mar 5;360(10):973-84.
39. Nair P, Pizzichini MM, Kjarsgaard M, Inman MD, Efthimiadis A, Pizzichini E, et al. Mepolizumab for prednisone-dependent asthma with sputum eosinophilia. N Engl J Med. 2009 Mar 5;360(10):985-93.
40. Pavord ID, Korn S, Howarth P, Bleecker ER, Buhl R, Keene ON, et al. Mepolizumab for severe eosinophilic asthma (DREAM): a multicentre, double-blind, placebo-controlled trial. Lancet. 2012 Aug 18;380(9842):651-9.
41. Pelaia G, Vatrella A, Maselli R. The potential of biologics for the treatment of asthma. Nat Rev Drug Discov. 2012 Dec;11(12):958-72.
42. Corren J, Lemanske RF, Hanania NA, Korenblat PE, Parsey MV, Arron JR, et al. Lebrikizumab treatment in adults with asthma. N Engl J Med. 2011 Sep 22;365(12):1088-98.
43. Gupta S, Siddiqui S, Haldar P, Entwisle JJ, Mawby D, Wardlaw AJ, et al. Quantitative analysis of high-resolution computed tomogrphy scans in severe asthma subphenotypes. Thorax. 2010;65(9):775-81.
44. Gupta S, Raj V, Castro M, Brightling CE Imaging in severe asthma. European Respiratory Monograph 2011; 12: 160-172
45. Guckel C, Wells AU, Taylor DA, Chabat F, Hansell DM: Mechanism of mosaic attenuation of the lungs on computed tomography in induced bronchospasm. J Appl Physiol 1999, 86(2):701- 708.
46. Tanaka N, Matsumoto T, Miura G, Emoto T, Matsunaga N, Ueda K, et al. Air trapping at CT: high prevalence in asymptomatic subjects with normal pulmonary function. Radiology 2003, 227(3):776-785.
47. Beigelman-Aubry C, Capderou A, Grenier PA, Straus C, Becquemin MH, Similowski T, et al. Mild intermittent asthma: CT assessment of bronchial cross-sectional area and lung attenuation at controlled lung volume. Radiology 2002, 223(1):181-187.
48. Busacker A, Newell JD Jr., Keefe T, Hoffman EA, Granroth JC, Castro M, et al. A multivariate analysis of risk factors for the air-trapping asthmatic phenotype as measured by quantitative CT analysis. Chest 2009, 135(1):48-56.
49. Gono H, Fujimoto K, Kawakami S, Kubo K Evaluation of airway wall thickness and air trapping by HRCT in asymptomatic asthma. Eur Respir J 2003, 22(6):965-971.
50. Newman KB, Lynch DA, Newman LS, Ellegood D, Newell JD Jr.: Quantitative computed tomography detects air trapping due to asthma. Chest 1994, 106(1):105-109.
51. Ueda T, Niimi A, Matsumoto H, Takemura M, Hirai T, Yamaguchi M, et al. Role of small airways in asthma: investigation using high-resolution computed tomography. J Allergy Clin Immunol 2006, 118(5):1019-1025.
52. Tunon-de-Lara JM, Laurent F, Giraud V, Perez T, Aguilaniu B, Meziane H, et al. Air trapping in mild and moderate asthma: effect of inhaled corticosteroids. J Allergy Clin Immunol 2007, 119(3):583-590.
53. Zeidler MR, Goldin JG, Kleerup EC, Kim HJ, Truong DA, Gjertson DW, et al. Small airways response to naturalistic cat allergen exposure in subjects with asthma. J Allergy Clin Immunol. 2006 Nov;118(5):1075-81.
54. Galbán CJ, Han MK, Boes JL, Chughtai KA, Meyer CR, Johnson TD, et al. Computed tomography-based biomarker provides unique signature for diagnosis of COPD phenotypes and disease progression. Nat Med. 2012;18(11):1711-5
55. Postma DS, Kerkhof M, Boezen HM, Koppelman GH. Asthma and chronic obstructive pulmonary disease: common genes, common environments? Am J Respir Crit Care Med. 2011 Jun 15;183(12):1588-94.
56. Undarmaa S, Mashimo Y, Hattori S, Shimojo N, Fujita K, Miyatake A, et al. Replication of genetic association studies in asthma and related phenotypes. J Hum Genet. 2010 Jun;55(6):342-9.
57. Moffatt MF, Gut IG, Demenais F, Strachan DP, Bouzigon E, Heath S, et al. GABRIEL Consortium. A large-scale, consortium-based genomewide association study of asthma. N Engl J Med. 2010 Sep 23;363(13):1211-21.
58. Torgerson DG, Ampleford EJ, Chiu GY, Gauderman WJ, Gignoux CR, Graves PE, et al. Meta- analysis of genome-wide association studies of asthma in ethnically diverse North American populations. Nat Genet. 2011 Jul 31;43(9):887-92.
59. Choy DF, Modrek B, Abbas AR, Kummerfeld S, Clark HF, Wu LC, et al. Gene expression patterns of Th2 inflammation and intercellular communication in asthmatic airways. J Immunol. 2011 Feb 1;186(3):1861-9.
60. Woodruff PG, Modrek B, Choy DF, Jia G, Abbas AR, Ellwanger A, et al. T-helper type 2- driven inflammation defines major subphenotypes of asthma. Am J Respir Crit Care Med. 2009 Sep 1;180(5):388-95.
61. Sridhar S, Schembri F, Zeskind J, Shah V, Gustafson AM, Steiling K, et al. Smoking-induced gene expression changes in the bronchial airway are reflected in nasal and buccal epithelium. BMC Genomics. 2008 May 30;9:259.
62. Jia CE, Zhang HP, Lv Y, Liang R, Jiang YQ, Powell H, et al. The Asthma Control Test and Asthma Control Questionnaire for assessing asthma control: Systematic review and meta- analysis. J Allergy Clin Immunol. 2013 Mar;131(3):695-703.
63. Schatz M, Sorkness CA, Li JT, Marcus P, Murray JJ, Nathan RA, et al: Asthma Control Test: reliability, validity, and responsiveness in patients not previously followed by asthma specialists. J Allergy Clin Immunol 2006, 117:549–556.
64. Juniper EF, Guyatt GH, Epstein RS, Ferrie PJ, Jaeschke R, Hiller TK. Evaluation of impairment of health-related quality of life in asthma: development of a questionnaire for use in clinical trials. Thorax 1992;47:76-83.
65. Herdman M, Gudex C, Lloyd A, Janssen M, Kind P, Parkin D, et al. Development and preliminary testing of the new five-level version of EQ-5D (EQ-5D-5L). Qual Life Res. 2011 Dec;20(10):1727-36.
66. Janssen MF, Pickard AS, Golicki D, Gudex C, Niewada M, Scalone L, et al. Measurement properties of the EQ-5D-5L compared to the EQ-5D-3L across eight patient groups: a multi- country study. Qual Life Res. 2012 Nov 25. [Epub ahead of print]
67. Riemersma R, Postma D, Kerstjens H, Buijssen K, Boezen M, Aalbers R, et al. Development of a questionnaire for the assessment of bronchial hyperresponsiveness. Prim Care Respir J. 2009 Dec;18(4):287-93.
68. Telenga ED, van den Berge M, Ten Hacken NH, Riemersma RA, van der Molen T, Postma DS. Small airways in asthma: their independent contribution to the severity of hyperresponsiveness. Eur Respir J. 2013 Mar;41(3):752-4.
69. Morisky DE, Ang A, Krousel-Wood M, Ward H. Predictive validity of a medication adherence measure for hypertension control. J Clin Hypertens 2008;10(5):348-54.
70. Moore WC, Meyers DA, Wenzel SE, Teague WG, Li H, Li X, et al. National Heart, Lung, and Blood Institute's Severe Asthma Research Program. Identification of asthma phenotypes using cluster analysis in the Severe Asthma Research Program. Am J Respir Crit Care Med. 2010 Feb 15;181(4):315-23.
71. Miller MR, Hankinson J, Brusasco V, Burgos F, Casaburi R, Coates A, et al. ATS/ERS Task Force. Standardisation of spirometry. Eur Respir J. 2005 Aug;26(2):319-38.
72. Quanjer PH, Stanojevic S, Cole TJ, Baur X, Hall GL, Culver BH, et al. Multi-Ethnic reference values for spirometry for the 3-95-yr age range: the global lung function 2012 equations. [Eur](http://www.ncbi.nlm.nih.gov/pubmed/22743675) [Respir J.](http://www.ncbi.nlm.nih.gov/pubmed/22743675) 2012 Dec;40(6):1324-43;
73. Oostveen E, MacLeod D, Lorino H, Farré R, Hantos Z, Desager K, et al. The forced oscillation technique in clinical practice: Methodology, recommendations and future developments. Eur Respir J 2003; 22:1026 –1041.
74. Robinson PD, Latzin P, Verbanck S, Hall GL, Horsley A, Gappa M, et al. Consensus statement for inert gas washout measurement using multiple- and single- breath tests. Eur Respir J. 2013 Mar;41(3):507-22.
75. Verbanck S, Schuermans D, Noppen M, Van Muylem A, Paiva M, Vincken W. Evidence of acinar airway involvement in asthma. Am J Respir Crit Care Med 1999;159:1545-50.
76. Crapo RO, Casaburi R, Coates AL, Enright PL, Hankinson JL, Irvin CG, et al. Guidelines for methacholine and exercise challenge testing-1999. This official statement of the American Thoracic Society was adopted by the ATS Board of Directors, July 1999. A m J Respir Crit Care Med. 2000 Jan;161(1):309-29.
77. Vollmer WM. Assessment of asthma control and severity. Ann Allergy Asthma Immunol 2004;93:409-14.
78. Juniper EF, Svensson K, Mörk AC, Ståhl E. Measurement properties and interpretation of three shortened versions of the asthma control questionnaire. Respir Med 2005;99:553-8.
79. Juniper EF, Guyatt GH, Cox FM, Ferrie PJ, King DR: Development and validation of the Mini Asthma Quality of Life Questionnaire. Eur Respir J 1999, 14:32–38.
80. Schiphof L, et al. Development Of A Tool To Recognize Small Airways Dysfunction In Asthma (SADT). Chapter DOI: 10.1164/ajrccm- conference.2013.187.1_MeetingAbstracts.A4220. Publication Date: 2013**.**
81. Hair, J. F. J., Anderson, R. E., Tatham, R. L., & Black, W. C. (1995). Multivariate data analysis (4th ed.). Saddle River, NJ: Prentice Hall in ;
82. Hogarty KY, Hines CV, Kromrey JD, Ferron JM and Mumford KR The Quality of Factor Solutions in Exploratory Factor Analysis: the Influence of Sample Size, Communality, and Overdetermination. Educational and Psychological Measurement 2005 65: 202;
83. Sim J and Wright CC**.** The Kappa Statistic in Reliability Studies: Use, Interpretation, and Sample Size Requirements. PHYS THER. 2005; 85:257-268;
84. Cantor, AB (1996). Sample Size Calculations for Cohen's Kappa, Psychological Methods, Vol 1, No. 2, pp 150 - 153.
